# Epigenetic modifier induced enhancement of fumiquinazoline C production in *Aspergillus fumigatus* (GA-L7): an endophytic fungus from *Grewia asiatica* L.

**DOI:** 10.1186/s13568-017-0343-z

**Published:** 2017-02-17

**Authors:** Ankita Magotra, Manjeet Kumar, Manoj Kushwaha, Praveen Awasthi, Chand Raina, Ajai Prakash Gupta, Bhahwal A. Shah, Sumit G. Gandhi, Asha Chaubey

**Affiliations:** 1 CSIR-Indian Institute of Integrative Medicine, Canal Road, Jammu, 180001 India; 2 Academy of Scientific & Innovative Research, New Delhi, 110001 India

**Keywords:** Endophyte, *Grewia asiatica* L., *Aspergillus fumigatus*, Epigenetic modifier, Valproic acid, Fumiquinazoline C

## Abstract

Present study relates to the effect of valproic acid, an epigenetic modifier on the metabolic profile of *Aspergillus fumigatus* (GA-L7), an endophytic fungus isolated from *Grewia asiatica* L. Seven secondary metabolites were isolated from *A. fumigatus* (GA-L7) which were identified as: pseurotin A, pseurotin D, pseurotin F2, fumagillin, tryprostatin C, gliotoxin and bis(methylthio)gliotoxin. Addition of valproic acid in the growth medium resulted in the alteration of secondary metabolic profile with an enhanced production of a metabolite, fumiquinazoline C by tenfolds. In order to assess the effect of valproic acid on the biosynthetic pathway of fumiquinazoline C, we studied the expression of the genes involved in its biosynthesis, both in the valproic acid treated and untreated control culture. The results revealed that all the genes i.e. Afua_6g 12040, Afua_6g 12050, Afua_6g 12060, Afua_6g 12070 and Afua_6g 12080, involved in the biosynthesis of fumiquinazoline C were overexpressed significantly by 7.5, 8.8, 3.4, 5.6 and 2.1 folds respectively, resulting in overall enhancement of fumiquinazoline C production by about tenfolds.

**Electronic supplementary material:**

The online version of this article (doi:10.1186/s13568-017-0343-z) contains supplementary material, which is available to authorized users.

## Introduction

Fungal endophytes produce a myriad bioactive class of compounds like alkaloids, terpenoids, steroids, benzopyranones, chinones, flavonoids, phenolic acids, quinones, tetralones, xanthones, and long chain peptides with a wide range of activities including anti-microbial, anticancer, immunosuppressant etc. (Nalli et al. 2015; Zhao et al. 2011). But the major concern is that their biosynthetic potential is much greater than that observed by fermentation, since a significant number of biosynthetic pathways remain dormant or under-expressed in conventional culture conditions (Cichewicz 2010). These dormant gene clusters can be expressed using molecular based approach (Gross 2009; Hertweck 2009) or cultivation based approach (Pettit 2011). Cultivation based approach include alteration in cultivation conditions such as change in media composition, pH, temperature, aeration, addition of elicitors, co-culturing, UV mutation, change in the type of fermentation, epigenetic modification etc. (Pettit 2011). Recent studies revealed that tickling the genome of fungi using epigenetic modifiers such as histone deacetylase (HDAC) and DNA methyltransferase (DNMT) can result in expression of the dormant biosynthetic gene clusters which alters the metabolic profile and hence results in the production of bioactives that were not produced under normal growth conditions (Menendez et al. 2016).

Epigenetic modifiers bring changes in gene expression without any alteration in DNA sequence of fungi (Xiao et al. 2013). Epigenetic modifications using chemical inhibitors (more specifically, HDAC or DNMT inhibitors) or inducers are found to be effective in stimulating the transcription of attenuated or silenced biosynthetic gene clusters, thereby resulting in the production or enhancement of a variety of secondary metabolites (Cichewicz 2010) e.g. *Cladosporium cladosporioides* when treated with HDAC inhibitor suberoylanilide hydroxamic acid (SAHA), led to the isolation of new cladochromes F, cladochromes G and calphostin B which was the first report of its co-occurrence with perylenequinones from a single source (Williams et al. 2008). In another attempt, addition of 310 μM of SAHA in *Aspergillus niger* resulted in the isolation of nygerone A, having a unique 1-phenylpyridin-4(1*H*)-one core skeleton (Henrikson et al. 2009). *Aspergillus fumigatus* has been recognized as a potential source of bioactive molecules (Shukla et al. 2014). A novel anticancer pro-drug deoxypodophyllotoxin has been isolated from *A. fumigatus* (Kusari et al. 2009). Also bioactive molecules like 12*β*-hydroxy-13*α*-methoxyverruculogen TR-2, fumitremorgin B, verruculogen, and helvolic acid have been isolated which showed potent antifungal activity (Li et al. 2012).

We have studied the effect of an epigenetic modifier, valproic acid on *A. fumigatus* (GA-L7), an endophyte isolated from *Grewia asiatica* L., from which seven compounds were isolated under normal growth conditions. Addition of valproic acid in the culture medium altered the metabolic profile of *A. fumigatus* (GA-L7) with enhancement of fumiquinazoline C production by ten folds, which was produced in trace amounts under normal cultivation conditions.

**Fig. 1:**
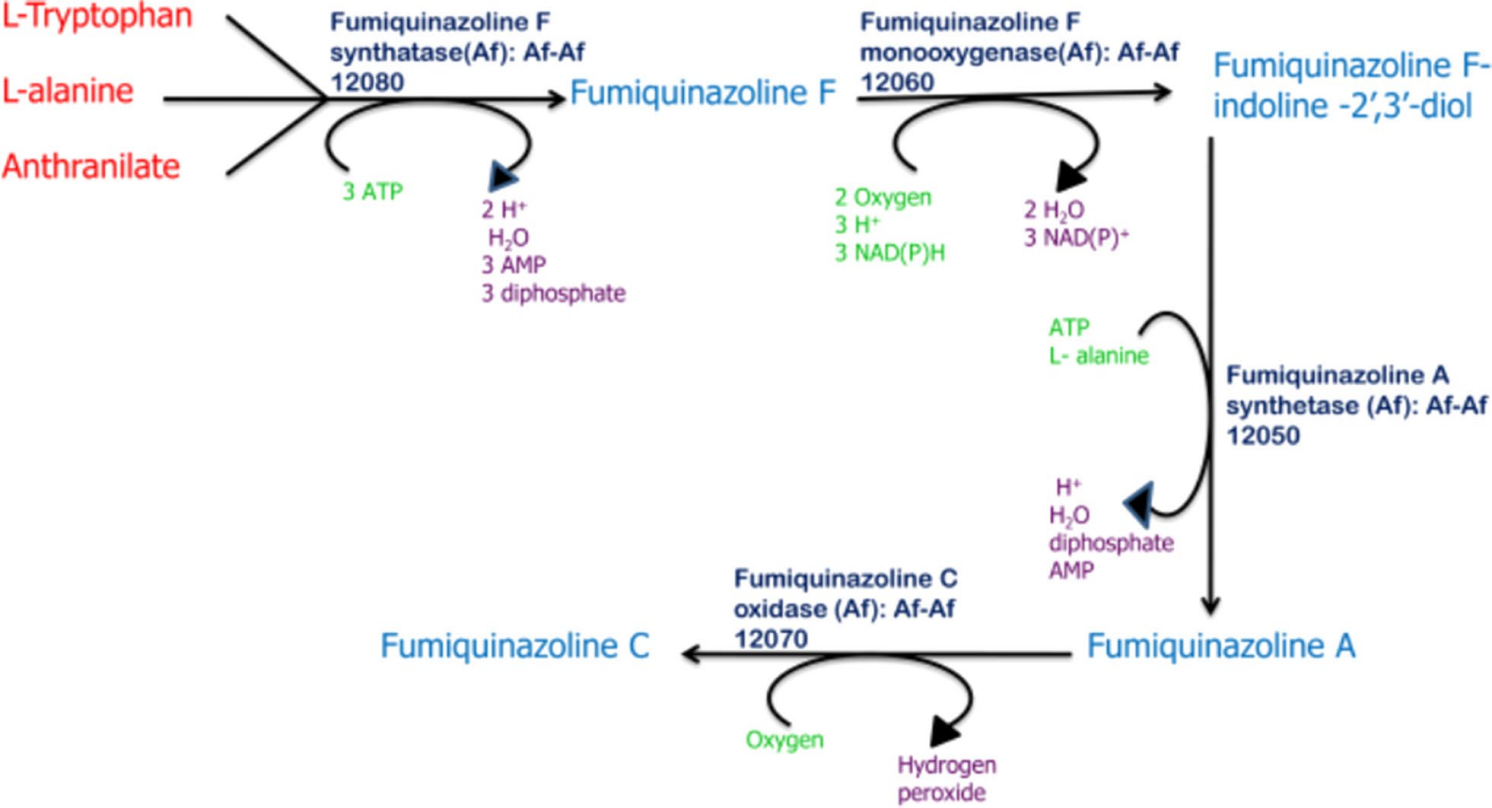
Schematic representation of the genes involved in the biosynthesis of fumiquinazoline C

Fumiquinazolines are peptidyl alkaloids and are reported to have significant antibacterial (Silva et al. 2004), antifungal (Belofsky et al. 2000) and antitumour properties (Han et al. 2007). Formation of fumiquinazoline C involves one unit of l-tryptophan, two units of l-alanine and one non proteinogenic amino acid i.e. l-anthranilate as precursors. As shown in Fig. 1, all the precursors are assembled by a trimodular NRPS Afua_6g 12080 to form fumiquinazoline F which further converts to fumiquinazoline A by the coordinated action of Afua_6g 12060 and Afua_6g 12050. Conversion of fumiquinazoline A to fumiquinazoline C is finally mediated by a mono-covalent flavoprotein Afua_6g 12070 (Ames et al. 2011). Therefore, in order to study, how valproic acid affects fumiquinazoline C biosynthetic genes, their expression profiles were studied in valproic acid treated culture vis-à-vis under normal cultivation conditions.

## Materials and methods

### Apparatus and reagents

Potato dextrose broth and agar were procured from Himedia Laboratories, India. Valproic acid was purchased from Alfa Aesar, Thermo Fischer Scientific, USA. Reagents and solvents used were LR grade and purchased from Fischer Scientific, USA. Silica gel coated aluminium plates from M/s Merck were used for thin liquid chromatography (TLC). Melting points (MPs) were measured in a Buchi-510 apparatus. ^1^H and ^13^C NMR spectra in CDCl_3_ were recorded on Bruker ARX 400 and 500 MHz spectrometers with TMS as an internal standard. Chemical shifts are expressed in parts per million (*δ* ppm); *J* values are given in Hertz. HRMS was recorded on G6540-UHD LC/MS Q-TOF Agilent Technologies. Optical rotations were measured on Perkin-Elmer 241 polarimeter at 25 °C using sodium D light. A triple quadrupole mass spectrometer, Agilent 6410 (Agilent Technologies, USA), equipped with an electrospray ionization (ESI) source was used for LCMS analysis. LCMS-grade acetonitrile, water and formic acid, used in the study, were purchased from Merck, Germany. LC was carried out on an Agilent 1260 infinity (Agilent Technologies, USA). A Chromolith High Resolution RP-18e column (100 × 4.6 mm) from Merck, Germany was used. Reagents for RNA isolation, cDNA preparation and Real time PCR were procured from Invitrogen, Life technologies, Carlsbad, USA; Ambion^®^ TURBO, DNA-free™, Life technologies; Promega, Madison, USA; Thermo Scientific, USA and Hoffmann-La Roche, Switzerland.

### Microorganism and fermentation conditions

Fresh healthy leaves of the plant *G. asiatica* L. were collected in sterile polythene bags from the Shiwalik region, Jammu, India and were processed immediately. Isolation of endophyte was done using previously reported method with some modifications (Strobel and Daisy 2003). Leaves were first washed under running tap water in order to remove dirt etc. Surface sterilization of the leaves was done using immersion in 70% alcohol for 30 s followed by immersion in 2% sodium hypochlorite for 3 min. Following treatment with the mentioned sterlients, the leaves were washed repeatedly for 5–6 times with autoclaved distilled water to get rid of sterlients. The leaves were then cut into small segments with and without midrib using sterile forceps and blades. These segments were placed on water agar plates and were incubated at 28 °C. The plates were monitored on a regular basis for the appearance of any endophyte. After 5–6 days, mycelia were seen growing from a leaf segment. The mycelia was inoculated on PDA (Potato Dextrose Agar) plate, subcultured and finally preserved on PDA slants.

For identification, fungus was first inoculated in PDB (Potato dextrose broth) and incubated at 28 °C till growth appeared. The mycelia were then separated by centrifugation at 10,000×*g* at 4 °C for DNA isolation. DNA was isolated using CTAB method (Raeder and Broda 1985). The ITS region of the fungus was amplified using universal ITS primers i.e. ITS1 (5′-TCCGTAGGTGAACCTGCGG-3′) and ITS4 (5′-TCCTCCGCTTATTGATATGC-3′) following polymerase chain reaction. The amplified DNA was then eluted and purified after being loaded on agarose gel (1%) using Kit. The sequencing PCR of the eluted product was run followed by sequencing using ABI 3500XL Genetic Analyzer. The sequenced products were then aligned with the sequences in GenBank using BlastN program (Altschul et al. 1997). A phylogenetic tree was prepared using closely related sequences using MEGA 5 (Tamura et al. 2011). The evolutionary distances were computed using the Maximum Composite Likelihood method and are in the units of the number of base substitutions per site (Tamura et al. 2004). The analysis involved ten nucleotide sequences. Codon positions included were 1st + 2nd + 3rd + noncoding. All positions containing gaps and missing data were eliminated. There were a total of 596 positions in the final dataset. Evolutionary analyses were conducted in MEGA5 as described by Saitou and Nei (1987).

### Growth conditions, extraction and isolation of compounds

The endophyte GA-L7 from freshly subcultured slant was inoculated in potato dextrose broth for 2 days at 28 °C with constant shaking at 200 rpm. 2% of seed culture was used to inoculate 25 L of production medium in a 50 L fermentor. The culture was allowed to grow under following conditions: 0.5 vvm air, 0.33 bar pressure, 100 RPM agitation and 28 °C temperature. 500 mL sample was withdrawn every 2 days to evaluate the production of bioactives. Fermentation was continued for 12 days till optimum production was seen based on the TLC pattern.

Following termination of fermentation, 10% methanol (MeOH) was added to the broth and homogenized to disrupt fungal mycelia followed by addition of an equal volume of dichloromethane (DCM). The extraction was carried out using National Cancer Institute’s (NCI) protocol (McCloud 2010). Following shaking in separating funnel, the solvent layer was separated and concentrated using rotary evaporator to get crude extract. The process was repeated thrice for the maximum recovery of metabolites.

The crude extract obtained from above procedure was then subjected to column chromatography on silica gel using different solvents to isolate compounds. Various solvent concentrations and combinations at which the compounds were eluted from the column include (3% MeOH:CH_2_Cl_2_) for pseurotin A, (3% MeOH:CH_2_Cl_2_) for pseurotin D, (3% MeOH:CH_2_Cl_2_) for pseurotin F2, (2% MeOH:CH_2_Cl_2_) for fumagillin, (35% EtOAc:Hexane) for tryprostatin C, (40% EtOAc:Hexane) for gliotoxin and (35% EtOAc:Hexane) for bis(dimethylthio)gliotoxin. The structures of the compounds were confirmed by comparing ^1^H, ^13^C NMR and HR-ESI-MS data with literature.

### Effect of valproic acid on the metabolic profile of *A. fumigatus* (GA-L7)

To study the effect of epigenetic modifier, 500 µM of valproic acid was supplemented in 100 mL potato dextrose broth prepared in 250 mL Erlenmeyer flasks. Also a control flask containing only potato dextrose broth was taken with the above flask. Both the flasks were inoculated with *A. fumigatus* (GA-L7) seed culture and were incubated at 28 °C under shaking conditions. Following termination of fermentation and extract preparation using above mentioned protocol, TLC profiles of the both the extracts were compared in mobile phase containing 5% MeOH in DCM. A bright spot at Rf value of 0.70 was observed in valproic acid treated extract which was not visible in the extract of the culture grown without valproic acid. The intensity of the spot was brightest after 8 days of fermentation.

### Quantification of compounds

For quantification of various compounds in the crude extract of *A. fumigatus* (GA-L7), pseurotin A, pseurotin D, pseurotin F_2_, fumagillin, tryprostatin C, gliotoxin, bis(dimethylthio)gliotoxin and fumiquinazoline C were used as standard compounds. Purity of all the investigated compounds was confirmed by HPLC and it was ≥98.50%. A triple quadrupole mass spectrometer, Agilent 6410 equipped with an electrospray ionization (ESI) source was used for the analysis. Prior to its use, the instrument was adjusted to meet the acceptance specifications defined by the manufacturer. Full scan data was acquired by scanning from *m*/*z* 100–1000 in MS2 mode, using a cycle time of 0.5 s, with a step size of 0.1 µs. Both the quadrupoles (Q1 and Q3) were operated at unit resolution. For the detection of investigated compounds, following operating conditions were used: Source and Mode, ESI; Polarity, positive (±); Collision gas, nitrogen; Capillary voltage, ±4000 V; Ion source temperature, 300 °C; Gas flow, 10 L/min; Dwell time, 10 ms; Resolution (Q1 and Q3), unit.

For the quantification of isolated compounds, 1 mg/mL of crude extract was prepared in MeOH and subjected to LC-ESI-MS analysis. The LC was carried out on an Agilent 1260 infinity, equipped with a quaternary pump, online degasser, column heater, autosampler. A Chromolith High Resolution RP-18e column (Merck, Germany; 100 × 4.6 mm) was used and the column temperature was 30 °C. Elution was performed at the flow rate of 0.6 mL/min with 0.1% aqueous formic acid (A) and acetonitrile (B) as the mobile phase. Gradient elution was used as follows: *t* (min), acetonitrile (%): (0, 20), (15, 50), (20, 50), (22, 20), (25, 20). After completion of run, the gradient was set back to initial conditions and the system was allowed to equilibrate for 2 min. The LC-ESI-MS analysis of MeOH fraction in total ion current (TIC) mode showed eight major peaks with mass *m/z* 454.1 (at *t*_*R*_ 4.6 min), 454.2, (at *t*_R_ 6.4 min), *m/z* 417.1 (at *t*_R_ 7.5 min), 444.1 (at *t*_R_ 10.9 min), *m/z* 352.4 (at *t*_R_ 11.3 min), *m/z* 349.3 (at *t*_R_ 12.7 min), *m/z* 359.1 (at *t*_R_ 13.6 min) and 458.2 (at *t*_R_ 18.9 min). After analysing LC-ESI-MS chromatogram of crude extract sample with LC-ESI-MS chromatogram of pure reference standards through spiking experiment, these peaks were identified as pseurotin F_2_, pseurotin A, pseurotin D, fumiquinazoline C, tryprostatin C, gliotoxin, bis(methylthio)gliotoxin and fumagillin. The molecular ions [M + Na]^+^ and [M-H]^−^ of compounds which were predominantly generated were chosen for quantification through selective ion monitoring (SIM) principle, because maximum of the compounds were being detected with their sodium ion adduct as well as to increase the specificity of isolated compounds from highly dense microbial extract.

The calibration equation (Pseurotin A, Tryprostatin C, Fumagillin, Pseurotin F2, Bis(methylthio)gliotoxin, Gliotoxin, Pseurotin D and Fumiquinazoline C was obtained by plotting LC–MS peak area (y) versus the concentration (x, ng/mL) of calibrators as

y = 153.923300x + 272,594.541506 (R^2^ = 0.9895), y = 25.022826x + 61346.381622 (R^2^ = 0.996), y = 2.024651x + 1429.800587 (R2 = 0.99583), y = 6.842660x − 4907.033500 (R^2^ = 0.9882), y = 22.324181x + 20189.179282 (R^2^ = 0.9900), y = 6.263182x + 7593.513001 (R^2^ = 0.9874), y = 164.086560x − 8347.005996 (R^2^ = 0.9895) and y = 8.821825x + 10,689.958682 (R^2^ = 0.9921), respectively. The equation showed very good linearity over the range.

### RNA extraction and cDNA synthesis for expression studies

For RNA extraction and cDNA synthesis, previously described methodology by Awasthi et al. (2015) was followed. Total RNA was isolated from *A. fumigatus* GA-L7 using TRIzol reagent (Invitrogen, Life technologies, Carlsbad, USA). DNase treatment was given to 10 µg of RNA (Ambion^®^ TURBO, DNA-free™, Life technologies). According to manufacturer’s instructions, first strand cDNA synthesis was carried out using the ImProm-II™ Reverse transcription system (Promega, Madison, USA). For cDNA synthesis, random hexamer primers were used (Thermo Scientific, USA) and 1 µg of the DNase treated RNA sample was taken as template.

### Relative expression analysis using quantitative real time PCR (qPCR)

Since biosynthesis of fumiquinazoline C involves expression of the genes, *Afua_6g12040, Afua_6g12050, Afua_6g12060, Afua_6g12070, Afua_6g12080*, therefore expression analysis of these genes was carried out. Extraction of RNA and cDNA was carried out using the described method. Primers were designed using Light-Cycler probe design software 2.0 (Hoffmann-La Roche, Switzerland) and were tested using conventional end point PCR for single band amplification as given in Table 1.

**Table 1:** Real time primers used in the expression studies of various genes involved in the biosynthesis of fumiquinazoline C

S. no	Primer code	Primer sequence (5′→3′)	No. of base pairs	T_m_ (°C)	Use
1	AFGAPDH01-F	CACGAACGCTATCGCTC	17	53.4	Amplification of housekeeping GAPDH gene in qPCR study
2	AFGAPDH01-R	GCTGGGTATGATATTCTCCG	20	52.4	
3	Afua_12040F	CCGAGTCTCCCGTCTTCTA	19	55.4	Amplification of Afua_12040 gene
4	Afua_12040R	CACTGATTCAGACTGCTGT	19	52.1	
5	Afua_12050F	CGACTGGAACTGGGTGA	17	54.1	Amplification of Afua_12050 gene
6	Afua_12050R	AGACTATTGTCCTGGTGC	18	51.4	
7	Afua_12060F	ATACTTTGCCCGAGAAGC	18	52.4	Amplification of Afua_12060 gene
8	Afua_12060R	TCCGTCTTCGATTGCCTGA	19	56.2	
9	Afua_12070F	CTTCTGGGCTATCAGGG	17	51.7	Amplification of Afua_12070 gene
10	Afua_12070R	GATTCCATCAGATCGAATACCG	22	52.9	
11	Afua_12080F	GCCAGTAATGGACAGTGTAAG	21	53.1	Amplification of Afua_12080 gene
12	Afua_12080R	GGCAGCAGAATATCATGGTT	20	52.9	

Real time PCR was carried out using the LightCycler^®^ 96 Real time PCR System (Hoffmann-La Roche, Switzerland) using the previous method (Awasthi et al. 2015) with some modifications. Thermal cycling conditions for the qPCR were: preincubation at 95 °C for 10 min, followed by 45 cycles of three step amplification (95 °C for 10 s, 54 °C for 12 s and 72 °C for 25 s). The PCR was followed by a dissociation curve analysis (heating to 95 °C for 10 s at normal ramping, followed by slow heating to 97 °C for 1 s at reduced ramping rate of 0.2 °C/s) to ensure PCR reaction specificity. Each assay was carried out in triplicates and a non template negative control was included. Glyceraldehyde-3-phosphate dehydrogenase (GAPDH) was used as housekeeping internal control for normalization. The threshold cycle (C_t_) of the amplification curve was used for calculations. The relative expression level was analyzed using 2^−∆∆Ct^ method (Livak and Schmittgen 2001), where ∆∆ C_t_ = (C_t,__target_ − C_t GAPDH_) _treated_ − (C_t,__target_ − C_t GAPDH_) _control_.

### Accession number

The culture has been submitted to Sir R. N. Chopra, Microbial Resource Centre, Jammu, India with accession number MRCJ-140. The sequence has been submitted to NCBI with accession no KF888650.

## Results

### Isolation and identification of *A. fumigatus* (GA-L7) from *G. asiatica* L.

**Fig. 2:**
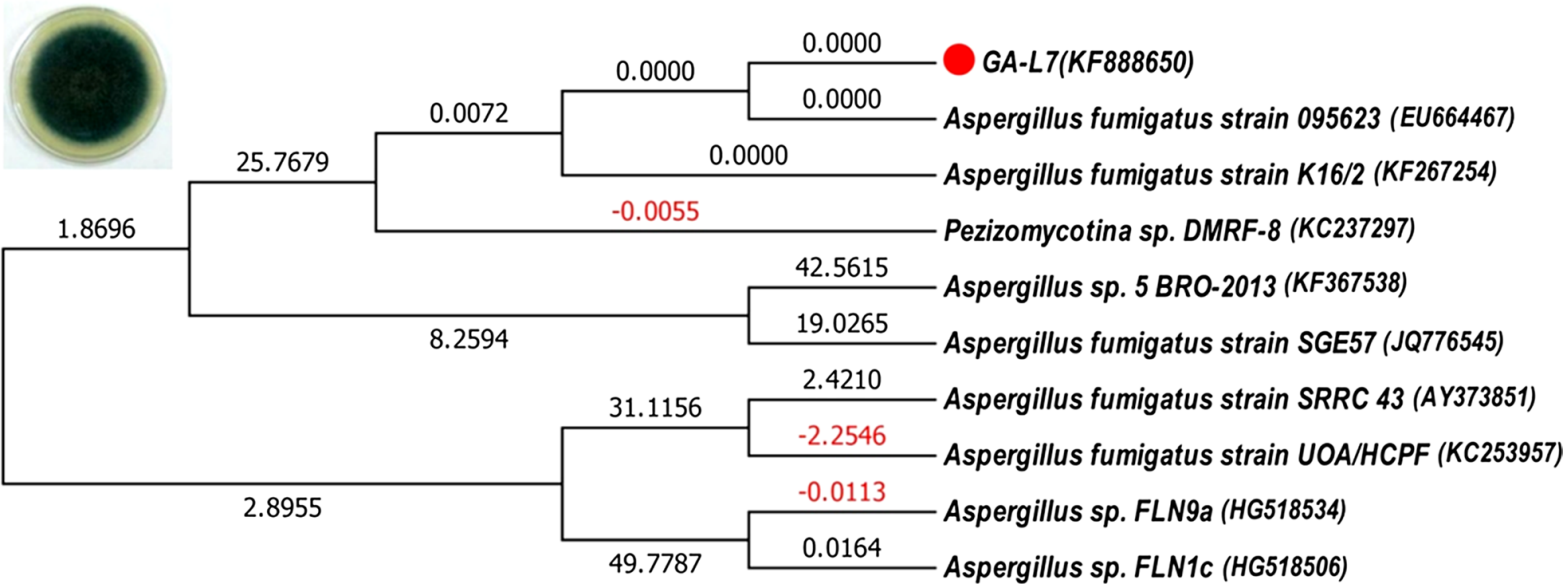
Evolutionary relationship of *Aspergillus fumigatus* (GA-L7). Phylogenetic tree was prepared using ITS rRNA gene sequences and the endophyte GA-L7 showed 100% homology with *Aspergillus fumigatus*

The culture plates bearing the leaf segments were observed regularly for appearance of any microbial growth. Fungal mycelia started appearing around the leaf segments on water agar plates after 5–6 days of incubation at 28 °C. It was then carefully transferred on PDA plates and finally preserved on PDA slants. Based on ITS rRNA gene sequencing, the endophyte GA-L7, used in the present study showed 100% homology with *A. fumigatus* and was found to be closest to *A. fumigatus* strain 095623 as shown in Fig. 2. The evolutionary history was inferred using the Neighbor-Joining method (Saitou and Nei 1987). The optimal tree with the sum of branch length = 181.44778756 is shown (next to the branches) in the Fig. 2.

### Isolation and characterization of secondary metabolites from *A. fumigatus* (GA-L7)

**Fig. 3:**
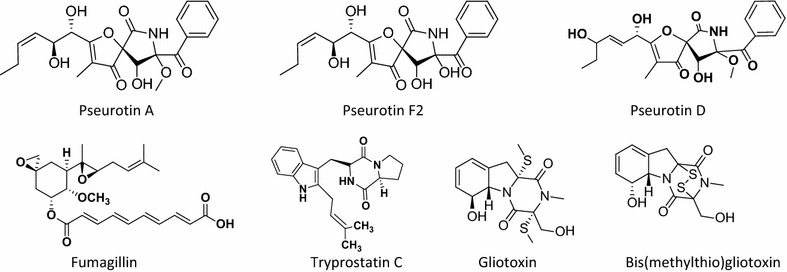
Compounds isolated from endophyte *Aspergillus fumigatus* (GA-L7) under normal culture conditions

**Fig. 4:**
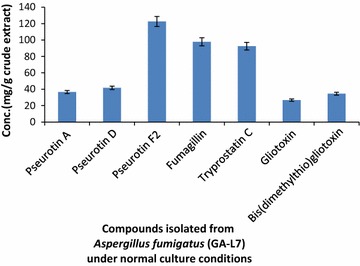
Quantification of compounds isolated from endophyte *Aspergillus fumigatus* (GA-L7) under normal culture conditions

Dichloromethane extract of *A. fumigatus* (GA-L7) was prepared using NCI protocol as described in experimental section. The structures of the isolated compounds were confirmed by comparing ^1^H, ^13^C NMR and HR-ESI-MS data with literature (Additional file 1: Figure S1). Seven compounds were isolated from the crude extract of *A. fumigatus* (GA-L7) which were identified as pseurotin A, pseurotin D, pseurotin F_2_, fumagillin, tryprostatin C, gliotoxin, and bis(dimethylthio)gliotoxin (Fig. 3) and their quantification was done using LC-ESI-MS as shown in Fig. 4. Based on quantification of the isolated compounds in the crude extract, it was found that pseurotin F2 (122.6 µg/mg crude extract) was present in maximum quantity in the crude extract of *A. fumigatus* (GA-L7) followed by fumagillin (97.9 µg/mg), tryprostatin C (92.5 µg/mg), pseurotin D (41.8 µg/mg), pseurotin A (36.6 µg/mg) and bis(dimethylthio)gliotoxin (34.5 µg/mg). Gliotoxin was present in minimum quantity (26.71 µg/mg crude extract).

### Effect of epigenetic modifiers on *A. fumigatus* (GA-L7)

**Fig. 5:**
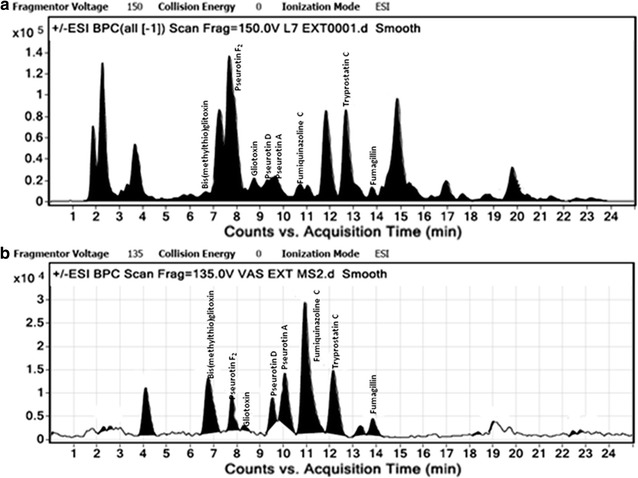
LC-ESI-MS graphs of extract from **a** control untreated *Aspergillus fumigatus* (GA-L7) and **b** valproic acid treated *Aspergillus fumigatus* (GA-L7)

**Fig. 6:**
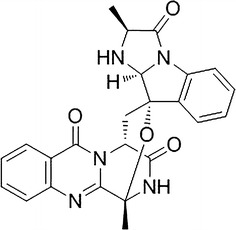
Structure of fumiquinazoline C isolated from valproic acid treated *Aspergillus fumigatus* (GA-L7)

Crude extract obtained from both valproic acid treated *A. fumigatus* (L7VA) and untreated *A. fumigatus* grown under normal conditions (L7C) was evaluated by respective TLC as well as LC-ESI-MS profiling. Both the TLC as well as the LC-ESI-MS pattern of crude extract obtained from valproic acid treated *A. fumigatus* (GA-L7) was observed to be different as compared to that of the extract prepared from culture grown under normal conditions. TLC profile of the extract from the valproic acid treated culture showed the presence of a UV visible spot at Rf value of 0.70 in mobile phase containing 5% MeOH in DCM which was not visible in the extract of the culture grown without valproic acid. Also, LC-ESI-MS profile of valproic acid treated extract (Fig. 5) showed an enhancement in the production of a secondary metabolite as an intense peak at retention time 10.9 min with *m/z* of 444.1 [M + H]^+^ was seen, which was earlier present in traces when culture was grown without valproic acid. The compound was isolated from the extract of valproic acid treated culture broth and it was further assigned the molecular formula C_24_H_21_N_5_O_4_ by HR-ESI-MS data at *m/z* of 444.2773 (calculated [M + H]^+^ 444.2754). Isolated molecule was then subjected to ^1^H and ^13^C NMR which led to the identification of compound as fumiquinazoline C (Fig. 6).

**Fig. 7:**
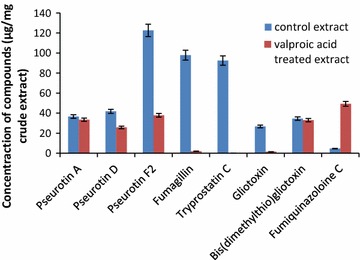
Quantification of compounds in extract prepared from valproic acid treated culture with respect to compounds present in crude extract of untreated control culture

In order to quantify the compounds, samples of crude extracts (1 mg/mL) obtained from valproic acid treated and untreated cultures were prepared in MeOH and subjected to LC-ESI-MS analysis (Fig. 7). It was observed that production of all the compounds decreased in the valproic acid treated extract except fumiquinazoline C, where it was increased by about tenfolds. As shown in Fig. 7, only 4.61 µg fumiquinazoline C per mg of crude extract was obtained from the culture grown without valproic acid, whereas it enhanced to 49.30 µg/mg crude extract when the culture was grown in presence of valproic acid. Pseurotin F2 showed a decrease in the production from 122.56 µg/mg crude extract in the control extract to 37.80 µg/mg crude extract in the valproic acid treated extract. There was a sharp decline in the fumagillin production from 97.86 µg/mg crude extract in the culture grown without valproic acid to 1.9 µg/mg crude extract in the valproic acid treated culture. Gliotoxin production also decreased from 26.7 µg/mg crude extract in the control extract to 1.49 µg/mg crude extract in the valproic acid treated extract.

### Relative expression analysis using quantitative real time PCR (qPCR)

**Fig. 8:**
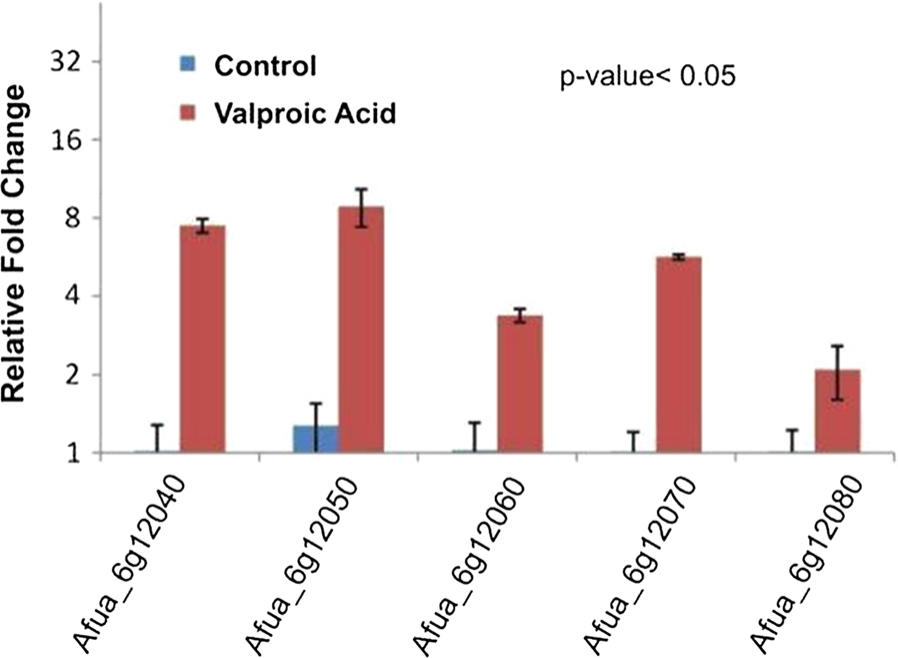
Expression profiling of the genes involved in fumiquinazoline C biosynthesis in *Aspergillus fumigatus* (GA-L7), an endophyte from *Grewia asiatica L*. All the genes exhibited significant upregulation in valproic acid treated culture compared to that of untreated control culture. GAPDH was used as an internal control. Relative quantification was carried out by taking the expression of the gene of interest in the untreated control culture as baseline for calculating fold change

Expression profiles of five genes involved in the biosynthesis of fumiquinazoline C was studied, in response to epigenetic modifier valproic acid treatment. GAPDH (glyceraldehyde phosphate dehydrogenase) was used as a housekeeping gene and relative quantification was carried out by taking the expression of the gene of interest in the untreated control culture as baseline for calculating fold change. All the genes involved in the formation of fumiquinazoline C got upregulated. Expression of Afua_6g 12040, Afua_6g 12050, Afua_6g 12060, Afua_6g 12070 and Afua_6g 12080 were enhanced by 7.5, 8.8, 3.4, 5.6 and 2.1 times respectively (Fig. 8). These studies reaffirm our observations which were based on TLC and LC-ESI-MS following supplementation of valproic acid, a known HDAC inhibitor, in the fermentation medium.

## Discussion

*Grewia asiatica* L. is well reported for its diverse medicinal uses and different parts of the plant are known for various pharmacological activities. Leaves are reported to have antimicrobial, anticancer, antiemetic and antiplatelet activities; fruits possess anticancer, antioxidant, antihyperglycemic and radioprotective properties while stem possess analgesic and anti-inflammatory activities (Zia-Ul-Haq et al. 2013). Despite these significant pharmacological activities, the plant has not been yet explored for the production of bioactivities from its endophytes. In our continuous efforts for screening biologically active secondary metabolites from the plant endophyte, we investigated chemical constituents produced by its *A. fumigatus* (GA-L7), an endophyte from *G. asiatica* L. *Aspergillus fumigatus* is known to produce a vast plethora of bioactives. A number of secondary metabolites have been isolated from endophytic *A. fumigatus* (Liu et al. 2004; Xie et al. 2015; Elfita et al. 2011). During present work, we have isolated and quantified seven secondary metabolites (Fig. 3) from endophytic *A. fumigatus* (GA-L7). These compounds include pseurotin F2 (122.6 µg/mg), fumagillin (97.9 µg/mg), tryprostatin C (92.5 µg/mg), pseurotin D (41.8 µg/mg), pseurotin A (36.6 µg/mg), bis(dimethylthio)gliotoxin (34.5 µg/mg) and gliotoxin (26.71 µg/mg).

Microorganisms consist of biosynthetic gene clusters that regulate the production of secondary metabolites. In recent past, significant efforts have been made in enhancing chemical diversity of microbial culture. In this regard, a striking breakthrough in this field is the introduction of epigenetic modifiers which brings changes in gene expression without any alteration in DNA sequence. Epigenetic modifications using chemical inhibitors (mainly HDAC or DNMT inhibitors) or inducers are found to be effective in stimulating the transcription of attenuated gene clusters, therefore resulting in the production of a variety of novel secondary metabolites or enhancement in the production of secondary metabolites (Yang et al. 2014). SAHA as an epigenetic modifier resulted in the modification of gene expression in *Chaetomium indicum* and six novel chaetophenols having a wide range of structural diversity were isolated (Asai et al. 2013). DNMT inhibitor such as 5-azacytidne also plays a significant role in epigenetic modification. 5-Azacytidine has been employed in *Diatrype* sp., where it induced the significant enhancement in secondary metabolite production and led to the isolation of two new polyketides. *Penicillium citreonigrum* when treated with 5-azacytidine resulted in enrichment of secondary metabolites in fungal culture (Henrikson et al. 2009).

In the present study, effect of three different epigenetic modifiers i.e. valproic acid, sodium butyrate and suberoylanilide hydroxamic acid (SAHA) on the metabolic profile of *A. fumigatus* (GA-L7) was studied. A significant variation in the metabolome of the strain was observed when treated with valproic acid, an HDAC inhibitor. Comparative profiling with LC-ESI-MS and TLC demonstrated that valproic acid treated *A. fumigatus* (GA-L7) showed an altered metabolic profile. LC-ESI-MS spectra showed ten folds enhancement in the production of a secondary metabolite, fumiquinazoline C (49.30 µg/mg crude extract) which was earlier present in very less quantities (4.61 µg/mg crude extract) in the crude extract (Fig. 5). Fumiquinazoline C was then isolated from the valproic acid treated extract of *A. fumigatus* (GA-L7) (Fig. 6). It was also observed that the production of the rest of the compounds was decreased in the valproic acid treated extract. As shown in Fig. 7, pseurotin F2 showed a decrease in the production from 122.56 µg/mg crude extract in the control extract to 37.80 µg/mg crude extract in the valproic acid treated extract. There was a sharp decrease in the fumagillin production from 97.86 µg/mg crude extract in the culture grown without valproic acid to 1.9 µg/mg crude extract in the valproic acid treated culture. Gliotoxin production also decreased from 26.7 µg/mg crude extract in the control extract to 1.49 µg/mg crude extract of valproic acid treated culture. Similar observations on valproic acid were made by other researchers, e.g. a range of epigenetic modifiers were employed in *Nigrospora sphaerica* SS67, where valproic acid was found to increase the production of secondary metabolites (Lopes et al. 2012). In the same year De Felicio et al. (2012) reported that valproic acid in combination with procaine speed up the metabolism in *Xylariaceae* sp. strain. The culture was found to produce metabolites in just 7 days which it usually produced in 14 days without any elicitation. We also observed maximum production of fumiquinazoline C in 8 days compared to 12 days fermentation period.

Various genes i.e. Afua_6g 12040, Afua_6g 12050, Afua_6g 12060, Afua_6g 12070 and Afua_6g 12080 are involved in the biosynthesis of fumiquinazoline C (Fig. 1). To investigate the effect of valproic acid on biosynthetic genes involved in the formation of fumiquinazoline C, we studied the expression profiling of these biosynthetic genes, both in untreated control culture and valproic acid treated culture. Expression of Afua_6g 12040, Afua_6g 12050, Afua_6g 12060, Afua_6g 12070 and Afua_6g 12080 were enhanced by 7.5, 8.8, 3.4, 5.6 and 2.1 times respectively in the valproic acid treated culture (Fig. 8). Thus all the genes involved in the formation of fumiquinazoline C were upregulated in the presence of epigenetic modifier, valproic acid.

To conclude, with the use of epigenetic modifiers we can get enhanced production of compounds which are produced in trace amounts under standard laboratory conditions. Thus, use of epigenetic modifiers may help to gain access to a vast repertoire of compounds that are either not produced or produced in minute undetectable quantities under normal fermentation conditions. Treatment of the endophyte *A. fumigatus* (GA-L7) with a known epigenetic modifier, valproic acid led to a significant increase in the production of fumiquinazoline C, by tenfolds. Expression studies revealed that all the genes i.e. Afua_6g 12040, Afua_6g 12050, Afua_6g 12060, Afua_6g 12070 and Afua_6g 12080 involved in the biosynthesis of fumiquinazoline C were up-regulated in presence of valproic acid, hence validating the fact that epigenetic modifiers can tickle the biosynthetic pathway resulting in an alteration in the metabolic profile of the microorganisms. This methodology can be useful for other fungal cultures for isolation of natural products which could significantly aid in efficient drug discovery.

## Additional file

**Additional file 1: Figure S1.** ^1^H and ^13^C NMR spectra of the isolated compounds.

## Abbreviations

PDA: potato dextrose agar
PDB: potato dextrose broth
MeOH: methanol
DCM: dichloromethane
EtOAc: ethyl acetate
LC: liquid chromatography
TIC: total ion current
SIM: selective ion monitoring SIM
GAPDH: glyceraldehyde-3-phosphate dehydrogenase
VA: valproic acid
LC-ESI-MS: liquid chromatography electron spray ionization mass spectroscopy

## References

[CR1] Altschul SF, Madden TL, Schaffer AA, Zhang J, Zhang Z, Miller W, Lipman DJ (1997). Gapped BLAST and PSI-BLAST: a new generation of protein database search programs. Nucl Acids Res.

[CR2] Ames BD, Haynes SW, Gao X, Evans BS, Kelleher NL, Tang Y, Walsh CT (2011). Complexity generation in fungal peptidyl alkaloid biosynthesis: oxidation of fumiquinazoline A to the heptacyclic hemiaminal fumiquinazoline C by the flavoenzyme Af12070 from *Aspergillus fumigatus*. Biochemistry.

[CR3] Asai T, Yamamoto T, Shirata N, Taniguchi T, Monde K, Fujii I, Gomi K, Oshima Y (2013). Structurally diverse chaetophenol productions induced by chemically mediated epigenetic manipulation of fungal gene expression. Org Lett.

[CR4] Awasthi P, Mahajan V, Gupta AP, Rasool S, Bedi YS, Vishwakarma RA, Gandhi SG (2015). Plant omics: isolation, identification, and expression analysis of cytochrome P450 gene sequences from *Coleus forskohlii*. OMICS.

[CR5] Belofsky GN, Anguera M, Jensen PR, Fenical W, Kock M (2000). Oxepinamides A-C and fumiquinazolines H-I: bioactive metabolites from a marine isolate of a fungus of the genus *Acremonium*. Chemistry.

[CR6] Cichewicz RH (2010). Epigenome manipulation as a pathway to new natural product scaffolds and their congeners. Nat Prod Rep.

[CR7] De Felicio R, Almeida TL, Cunha ÉS, Tomaz JC, Debonsi HM (2012). Secondary metabolism variation in endophytic marine fungi by chemical epigenetic elicitation approaches. Planta Med.

[CR8] Elfita E, Muharni M, Indah T (2011). Secondary metabolite of *Aspergillus fumigatus*, endophytic fungi of the medicinal plant *Garcinia griffithii*. Makara Seri Sains.

[CR9] Gross H (2009). Genomic mining—a concept for the discovery of new bioactive natural products. Curr Opin Drug Discov Dev.

[CR10] Han XX, Xu XY, Cui CB, Gu QQ (2007). Alkaloidal compounds produced by a marine-derived fungus, *Aspergillus fumigatus* H1-04, and their antitumor activities. Chin J Med Chem.

[CR11] Henrikson JC, Hoover AR, Joyner PM, Cichewicz RH (2009). A chemical epigenetics approach for engineering the in situ biosynthesis of a cryptic natural product from *Aspergillus niger*. Org Biomol Chem.

[CR12] Hertweck C (2009). Hidden biosynthetic treasures brought to light. Nat Chem Biol.

[CR14] Kusari S, Lamshöft M, Spiteller M (2009). *Aspergillus fumigatus* Fresenius, an endophytic fungus from *Juniperus communis* L. Horstmann as a novel source of the anticancer pro-drug deoxypodophyllotoxin. J Appl Microbiol.

[CR15] Li XJ, Zhang Q, Zhang AL, Gao JM (2012). Metabolites from *Aspergillus fumigatus*, an endophytic fungus associated with *Melia azedarach*, and their antifungal, antifeedant, and toxic activities. J Agric Food Chem.

[CR16] Liu JY, Song YC, Zhang Z, Wang L, Guo ZJ, Zou WX, Tan RX (2004). *Aspergillus fumigatus* CY018, an endophytic fungus in *Cynodon dactylon* as a versatile producer of new and bioactive metabolites. J Biotechnol.

[CR17] Livak KJ, Schmittgen TD (2001). Analysis of relative gene expression data using real-time quantitative PCR and the 2(-ΔΔ(C(T)) method. Methods.

[CR18] Lopes AA, da Silva DB, Lopes NP, Pupo MT (2012). Epigenetic modulation changed the secondary metabolite profile in the endophyte *Nigrospora sphaerica* SS67. Planta Med.

[CR20] McCloud TG (2010). High throughput extraction of plant, marine and fungal specimens for preservation of biologically active molecules. Molecules.

[CR21] Menendez VZ, Bonilla MP, Victoria IP, Martin J, Munoz F, Reyes F, Tormo JR, Genilloud R (2016). Multicomponent analysis of differential induction of secondary metabolic profiles in fungal endophytes. Molecules.

[CR22] Nalli Y, Mirza DN, Wani ZA, Wadhwa B, Mallik FA, Raina C, Riyaz-Ul-Hassan S, Ali A (2015). Phialomustin A-D, new antimicrobial and cytotoxic metabolites from an endophytic fungus, *Phialophora mustea*. RSC Adv.

[CR23] Pettit RK (2011). Small-molecule elicitation of microbial secondary metabolites. Microbial Biotechnol.

[CR24] Raeder U, Broda P (1985). Rapid preparation of DNA from filamentous fungi. Lett Appl Microbiol.

[CR25] Saitou N, Nei M (1987). The neighbor-joining method: a new method for reconstructing phylogenetic trees. Mol Biol Evol.

[CR26] Shukla ST, Habbu PV, Kulkarni VH, Jagadish KS, Pandey AR, Sutariya VN (2014). Endophytic microbes: a novel source for biologically/pharmacologically active secondary metabolites. Asian J Pharmacol Toxicol.

[CR27] Silva MG, Furtado NA, Pupo MT, Fonseca MJ, Said S, da Silva Filho AA, Bastos JK (2004). Antibacterial activity from *Penicillium corylophilum* Dierckx. Microbiol Res.

[CR28] Strobel G, Daisy B (2003). Bioprospecting for microbial endophytes and their natural products. Microbiol Mol Biol Rev.

[CR29] Tamura K, Nei M, Kumar S (2004). Prospects for inferring very large phylogenies by using the neighbor-joining method. Proc Natl Acad Sci USA.

[CR30] Tamura K, Peterson D, Peterson N, Stecher G, Nei M, Kumar S (2011). MEGA5: molecular evolutionary genetics analysis using maximum likelihood, evolutionary distance, and maximum parsimony methods. Mol Biol Evol.

[CR31] Williams RB, Henrikson JC, Hoover AR, Lee AE, Cichewicz RH (2008). Epigenetic remodeling of the fungal secondary metabolome. Org Biomol Chem.

[CR32] Xiao L, Yin Y, Sun W, Zhang F, Li Z (2013). Enhanced production of (+)-terrein by *Aspergillus terreus* strain PF26 with epigenetic modifier suberoylanilide hydroxamic acid. Process Biochem.

[CR33] Xie F, Li XB, Zhou JC, Xu QQ, Wang XN, Yuan HQ, Lou HX (2015). Secondary metabolites from *Aspergillus fumigatus*, an endophytic fungus from the liverwort *Heteroscyphus tener* (Steph.) Schiffn. Chem Biodivers.

[CR34] Yang XL, Huang L, Ruan XL (2014). Epigenetic modifiers alter the secondary metabolite composition of a plant endophytic fungus, *Pestalotiopsis crassiuscula* obtained from the leaves of *Fragaria chiloensis*. J Asian Nat Prod Res.

[CR35] Zhao J, Shan T, Mou Y, Zhou L (2011). Plant-derived bioactive compounds produced by endophytic fungi. Mini Rev Med Chem.

[CR36] Zia-Ul-Haq M, Stanković MS, Rizwan K, Feo VD (2013). *Grewia asiatica* L., a food plant with multiple uses. Molecules.

