# Supplementary material for: Epigenetic modifier induced enhancement of fumiquinazoline C production in Aspergillus fumigatus (GA-L7): an endophytic fungus from Grewia asiatica L
Source: AMB Express. 2017 Feb 17;7:43. doi: 10.1186/s13568-017-0343-z (PMC5315648; doi:10.1186/s13568-017-0343-z)
Supplement: Supplementary file 1 — Additional file 1: Figure S1. 1H and 13C NMR spectra of the isolated compounds. [file 13568_2017_343_MOESM1_ESM.pdf]

Title Page

Epigenetic modifier induced enhancement of fumiquinazoline C production in *Aspergillus fumigatus* (GA-L7): an endophytic fungus from *Grewia asiatica* L.

Authors: Ankita Magotra[a,b], Manjeet Kumar[a], Manoj Kushwaha[a], Praveen Awasthi[a], Chand Raina[a], Ajai Prakash Gupta[a], Bhahwal A. Shah[a,b], Sumit G. Gandhi[a,b], and Asha Chaubey[a,b]

Affiliations: [a]*CSIR-Indian Institute of Integrative Medicine, Canal Road, Jammu,180001, INDIA*

[b]*Academy of Scientific & Innovative Research, New Delhi, 110001, India*

Corresponding authors:

Bhahwal A. Shah (email: bashah@iiim.ac.in, Ph: +91-191-2585006-13 Ext 311; Fax: +91-191-2569333

Sumit G. Gandhi (email:sumit@iiim.ac.in, Ph: +91-191-2585006-13 Ext 359; Fax: +91-191-2569333

Asha Chaubey (email: achaubey@iiim.ac.in, Ph: +91-191-2585006-13 Ext 239; Fax: +91-191-2569333

Supplementary information

Fig. S1: ^{1}H and ^{13}C NMR spectra of the isolated compounds

Pseurotin A

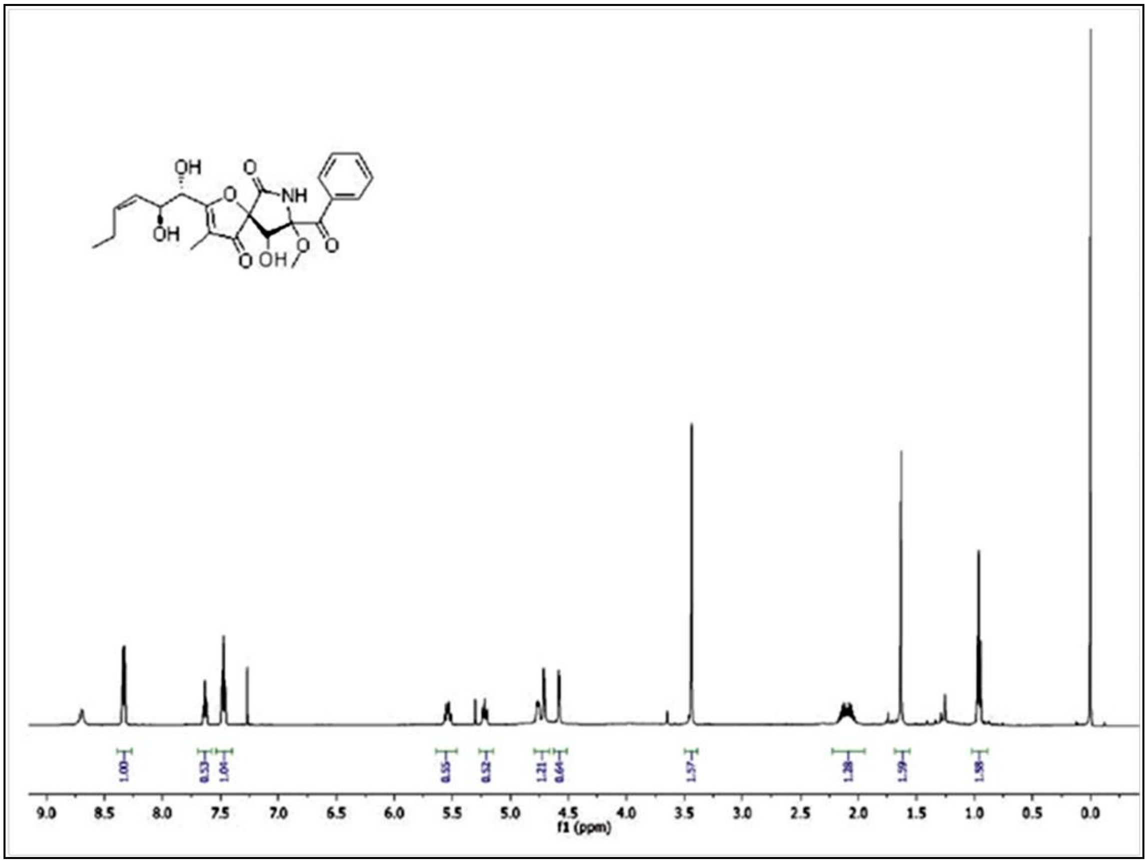

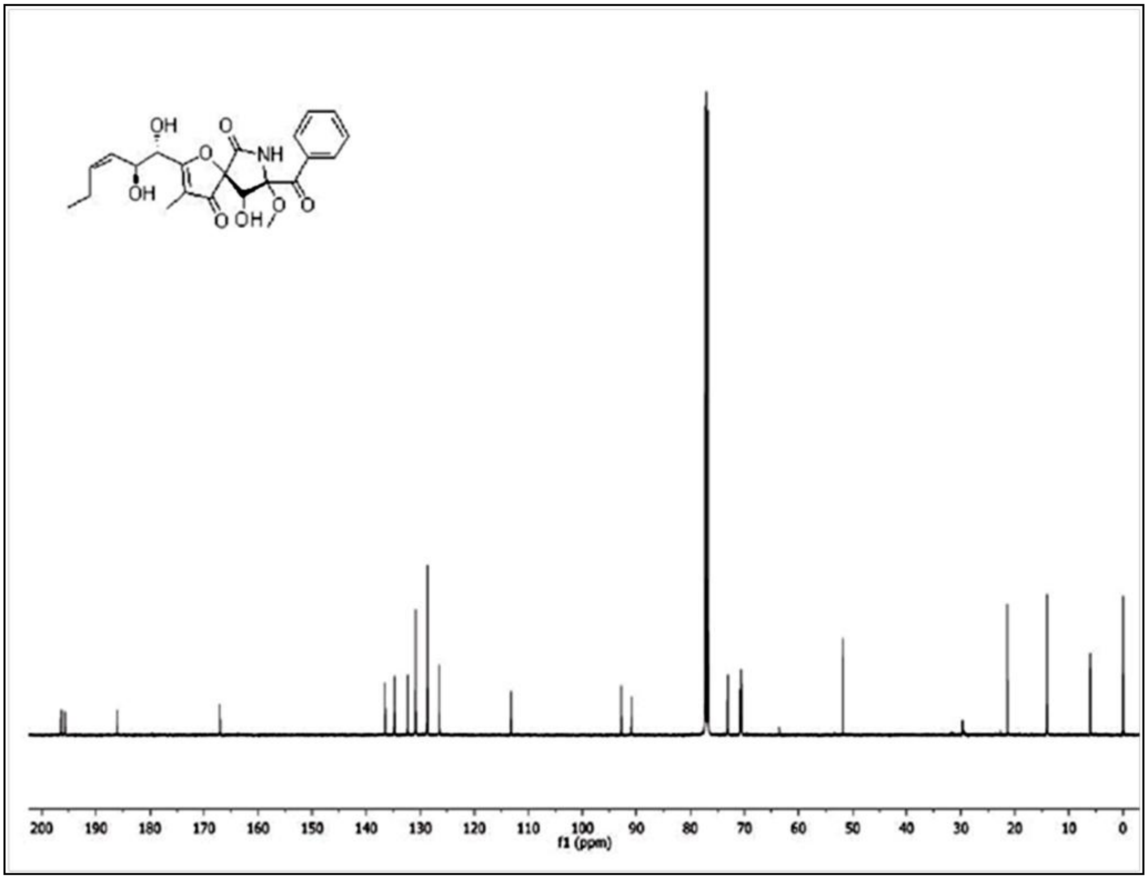

Pseurotin A : 3% MeOH:CH_2Cl_2, 100-2—silica gel, White crystalline solid, melting point- 163-165 ^{o}C; $[\alpha]^{25}_{D}$ -9.8 (*c* 1.0, CH_3OH);^{1}H NMR (500 MHz, $CDCl_3$) δ 8.33 (d, *J* = 7.6 Hz, 2H), 7.63 (t, *J* = 7.4, 1.0 Hz, 1H), 7.47 (t, *J* = 7.8, 1.1 Hz, 2H), 5.62 – 5.48 (m, 1H), 5.22 (t, *J* = 11, 9.9 Hz, 1H), 4.75 (dd, *J* = 14.5, 7.2 Hz, 1H), 4.71 (s, 1H), 4.58 (d, *J* = 4.3 Hz, 1H), 3.45 (s, 3H), 2.18 – 1.99 (m, 2H), 1.65 (s, 3H), 1.02 – 0.92 (m, 3H); ^{13}C NMR (125 MHz, $CDCl_3$) δ 196.4, 195.7, 186.0, 167.0, 136.5, 134.7, 132.3, 130.8, 128.6, 126.4, 113.1, 92.8, 90.9, 73.1, 70.8, 70.6, 51.8, 21.3, 14.1, 6.0.HR-ESI-MS (*m*/*z*) 454.1492 $[M+Na]^+$ (calculated for $[C_{22}H_{25}NO_8+Na]^+$ 454.1478).

Pseurotin D

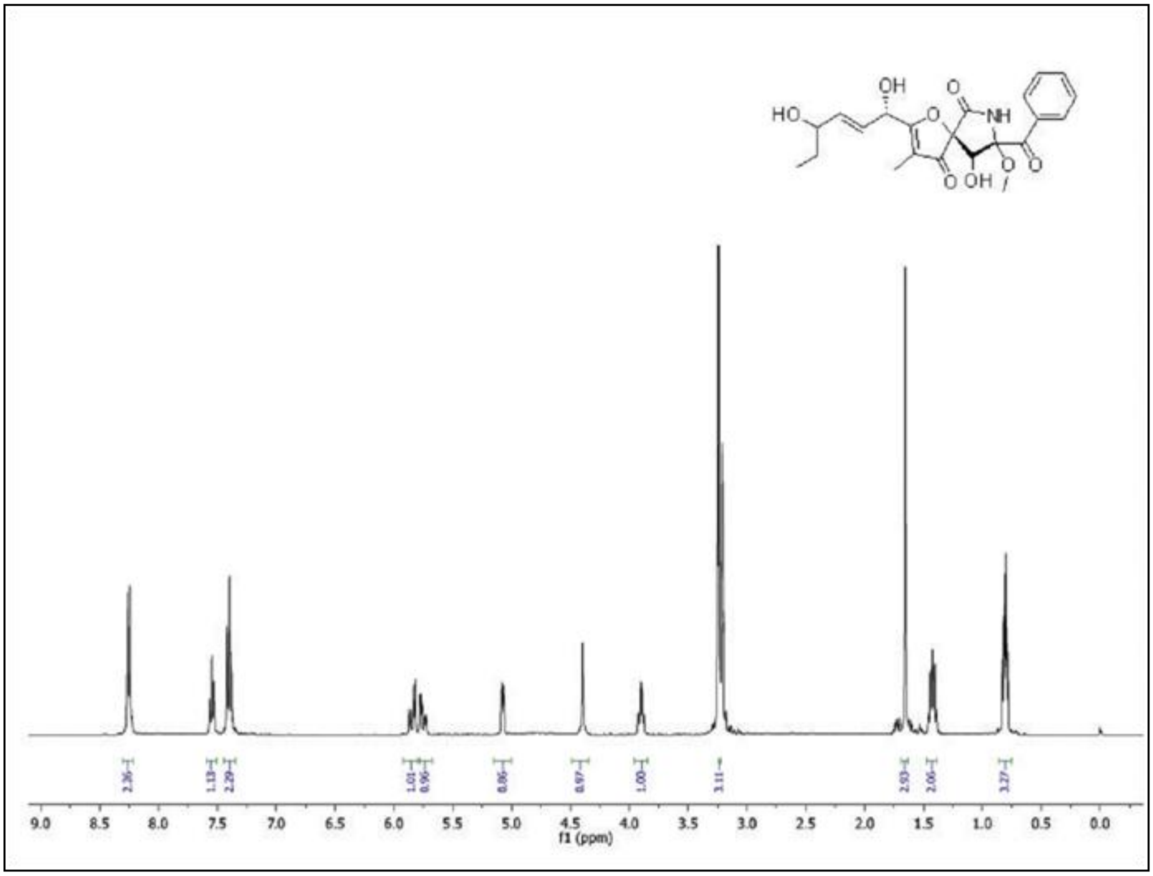

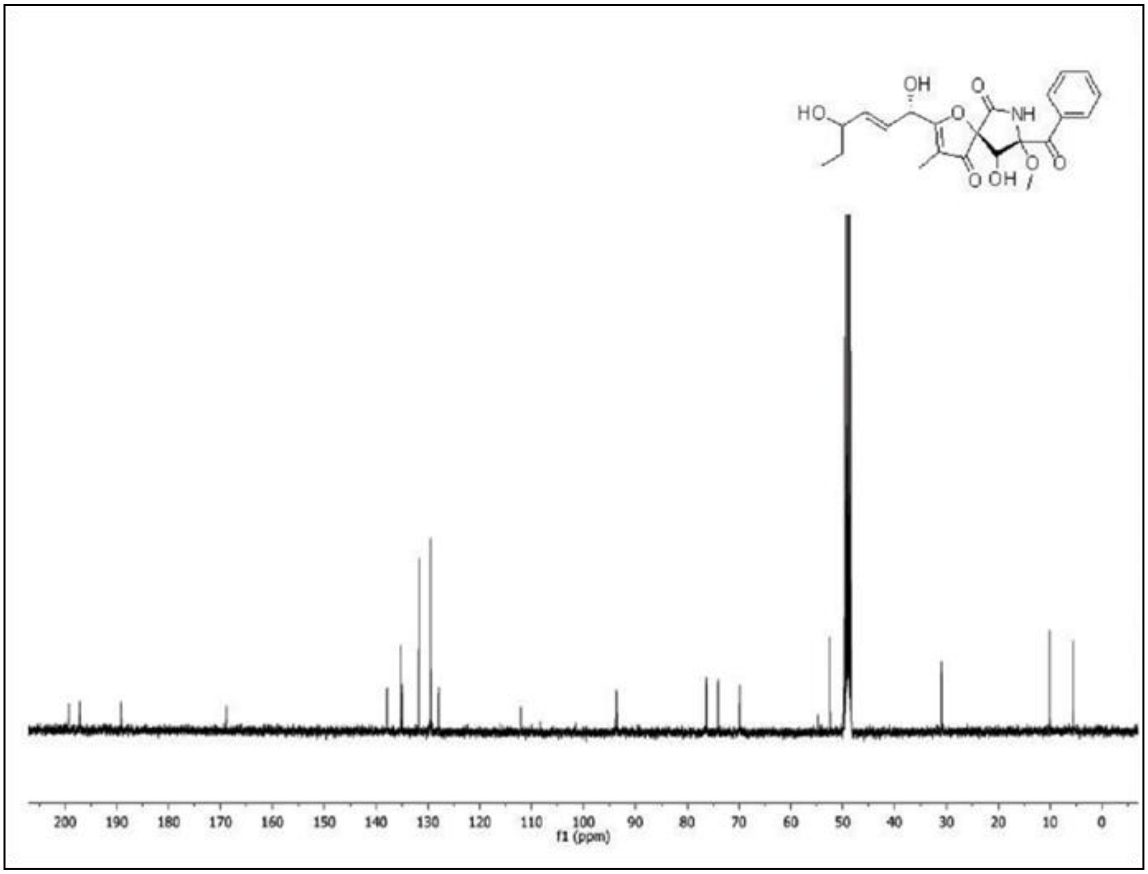

Pseurotin D: 3% MeOH:CH_2Cl_2 in100-200 silica gel for fractionation then prep TLC at 5% MeOH:CH_2Cl_2, Yellow semi-solid; $[\alpha]^{25}_{D}$ -0.7 (*c* 0.2, CH_3OH); ^{1}H NMR (400 MHz, MeOD) δ 8.24 (d, *J* = 7.6 Hz, 2H), 7.54 (m, 1H), 7.45 – 7.35 (m, 2H), 5.92 – 5.78 (m, 1H), 5.80 – 5.68 (m, 1H), 5.15 – 5.00 (m, 1H), 4.49 – 4.34 (m, 1H), 3.95 – 3.84 (m, 1H), 3.23 (s, 3H), 1.64 (s, 3H), 1.47 – 1.39 (m, 2H), 0.86 – 0.75 (m, 3H); ^{13}C NMR (100 MHz, MeOD) δ 199.2, 197.1, 189.0, 168.8, 137.9, 135.1, 134.9, 131.7, 129.5, 127.9, 112.1, 93.8, 93.5, 76.6, 73.9, 69.8, 52.4, 30.9, 10.0, 5.4; HR-ESI-MS (*m*/*z*) 454.1420 $[M+Na]^+$ (calculated for $[C_{22}H_{25}NO_8+Na]^+$ 454.1478).

Pseurotin F2

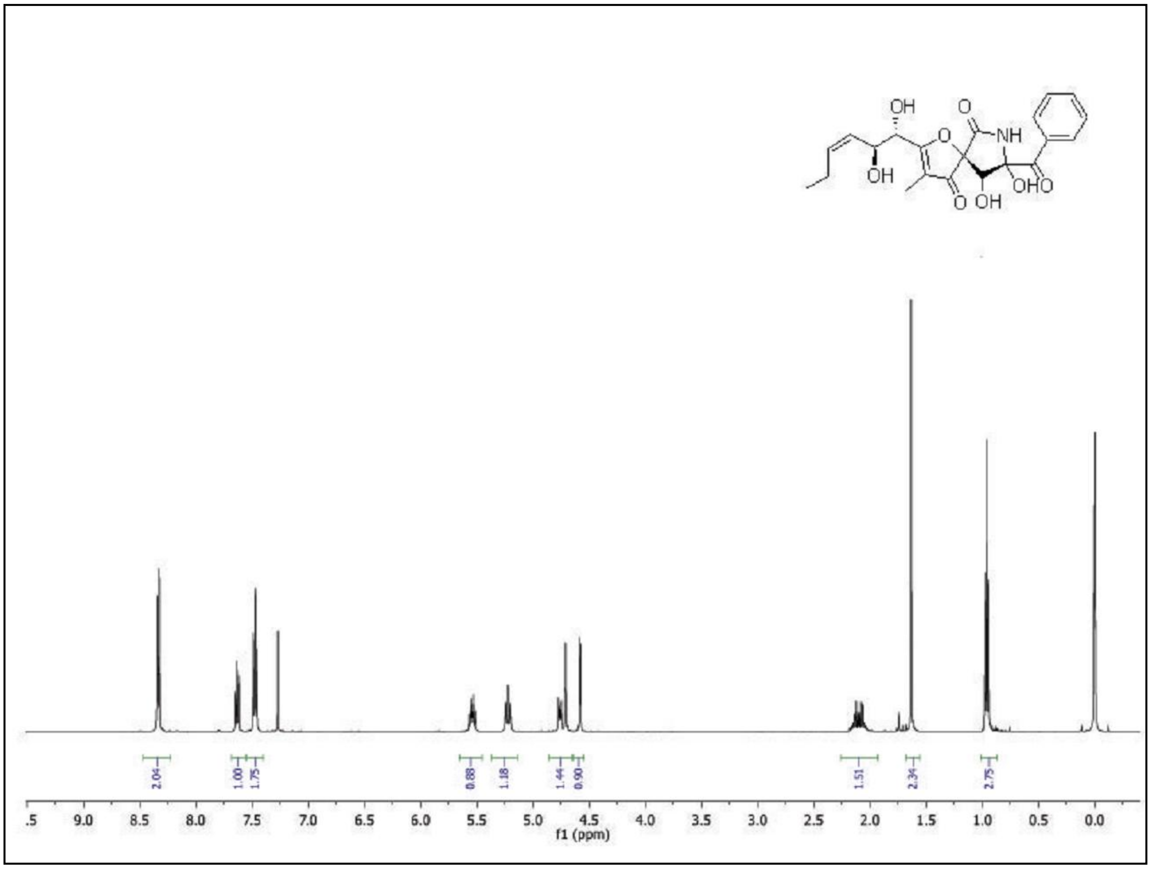

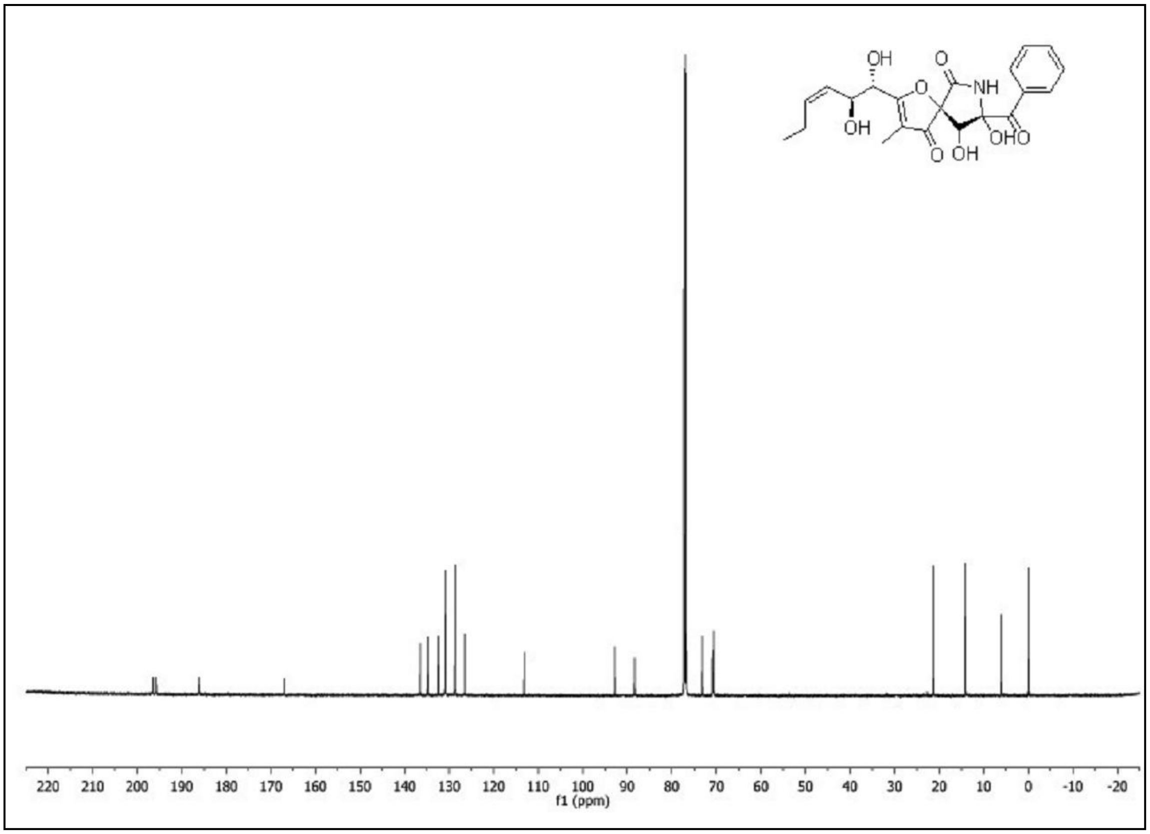

Pseurotin F_2 : 3% MeOH:CH_2Cl_2in100-200 silica gel for fractionation then prep TLC at 5% MeOH:CH_2Cl_2, Yellow semi-solid, $[\alpha]^{25}_D$ -12.7 (*c*0.2, CH_3OH);^{1}H NMR (500 MHz, $CDCl_3$) δ 8.32 (dd, *J* = 10.1, 8.8 Hz, 2H), 7.68 – 7.56 (m, 1H), 7.46 (dd, *J* = 15.9, 8.1 Hz, 2H), 5.65 – 5.45 (m, 1H), 5.37 – 5.14 (m, 1H), 4.86 – 4.65 (m, 2H), 4.59 (dd, *J* = 11.4, 4.6 Hz, 1H), 2.26 – 1.93 (m, 2H), 1.62 (s, 3H), 0.95 (s, 3H); ^{13}C NMR (125 MHz, $CDCl_3$) δ 196.1, 195.7, 186.2, 167.3, 136.8, 134.7, 132.3, 130.2, 128.5, 126.4, 112.9, 92.6, 87.9, 73.4, 70.8, 70.4, 21.3, 14.1, 6.0; HR-ESI-MS (*m/z*) 440.1325 $[M+Na]^+$, major m/z 416.1349 $[M-H]^-$ in negative mode, (calculated for $[C_{21}H_{23}NO_8+Na]^+$ 440.1321).

Fumagillin

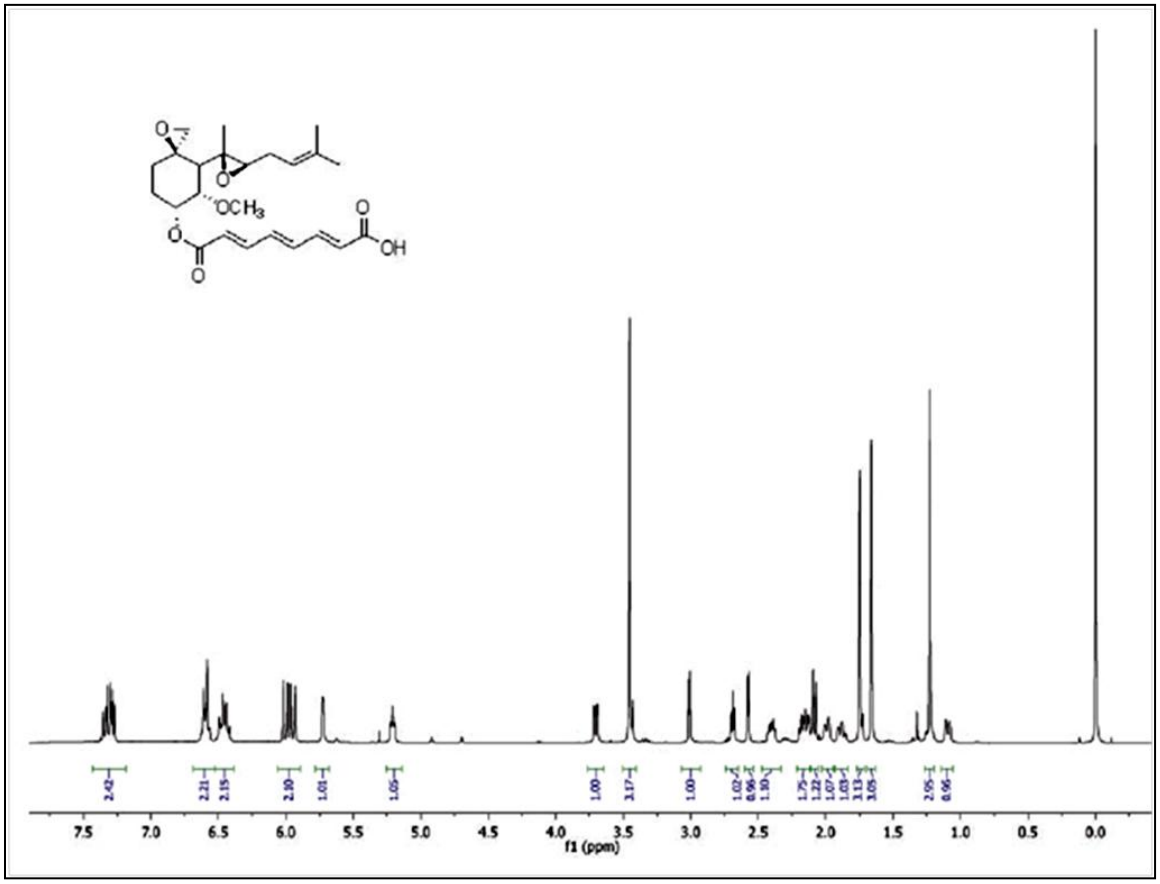

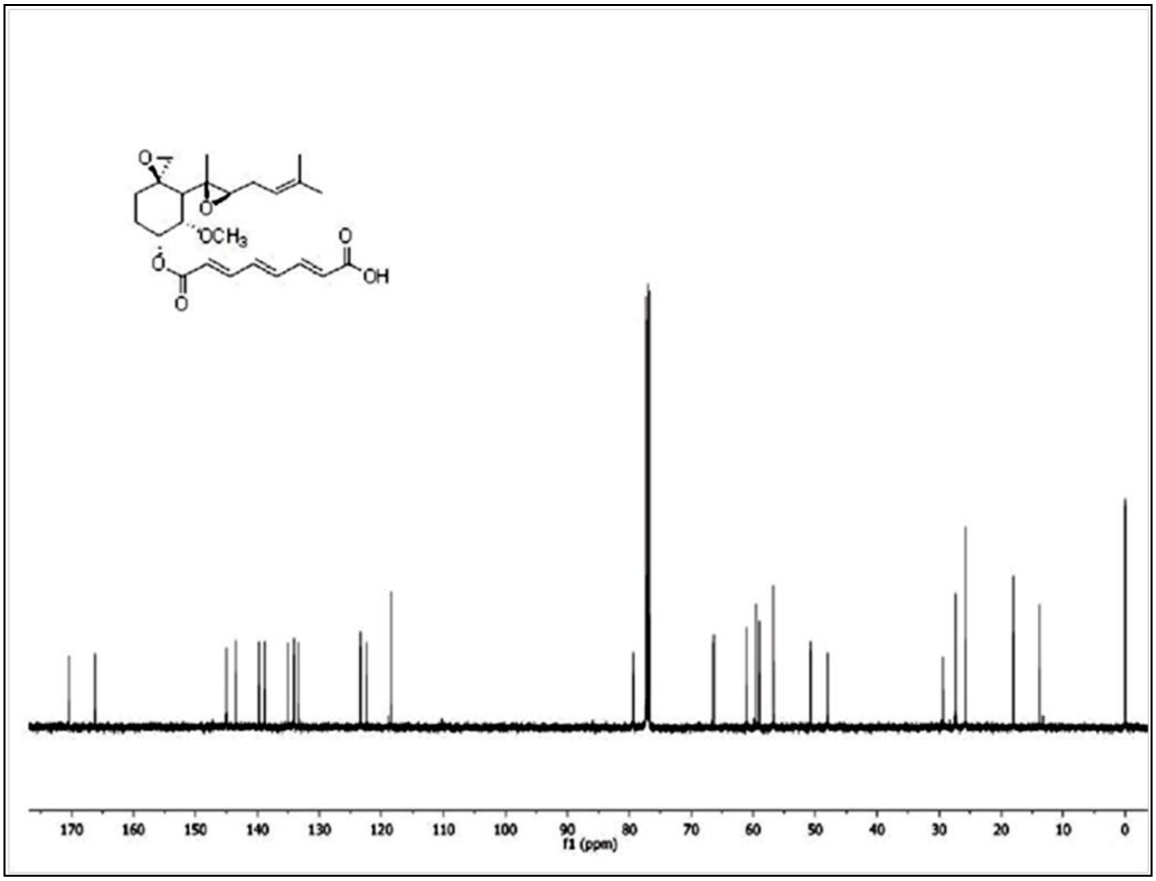

Fumagillin: 2% MeOH:CH_2Cl_2, 100-200 silica gel, Yellow solid, melting point- 193-194 °C; $[\alpha]^{25}_{D}$ -12.4 (*c* 0.5, CH_3OH); ^{1}H NMR (500 MHz, $CDCl_3$) δ 7.39 – 7.24 (m, 2H), 6.64 – 6.57 (m, 2H), 6.51 – 6.38 (m, 2H), 5.97 (dd, *J* = 27.7, 15.3 Hz, 2H), 5.21 (t, *J* = 7.4, 1.9 Hz, 1H), 3.70 (dd, *J* = 11.3, 2.7 Hz, 1H), 3.44 (s, 3H), 3.01 (d, *J* = 4.3 Hz, 1H), 2.75 – 2.64 (m, 1H), 2.57 (d, *J* = 4.3 Hz, 1H), 2.47 – 2.32 (m, 1H), 2.24 – 2.10 (m, 2H), 2.08-2.06 (m, 1H), 1.96-1.82 (m, 2H), 1.74 (s, 3H), 1.66 (s, 3H), 1.26 – 1.18 (s, 1H), 1.10 (m, 1H); ^{13}C NMR (125 MHz, $CDCl_3$) δ 170.3, 166.1, 145.0, 143.5, 139.7, 138.8, 135.0, 134.1, 133.3, 123.3, 122.4, 118.4, 79.3, 66.3, 61.0, 59.5, 59.0, 56.7, 50.8, 47.9, 29.3, 27.3, 25.7, 25.2, 18.0, 13.8;HR-ESI-MS (*m/z*) 481.2170 $[M+Na]^+$ (calculated for $[C_{26}H_{34}O_7+Na]^+$ 481.2202).

Tryprostatin C

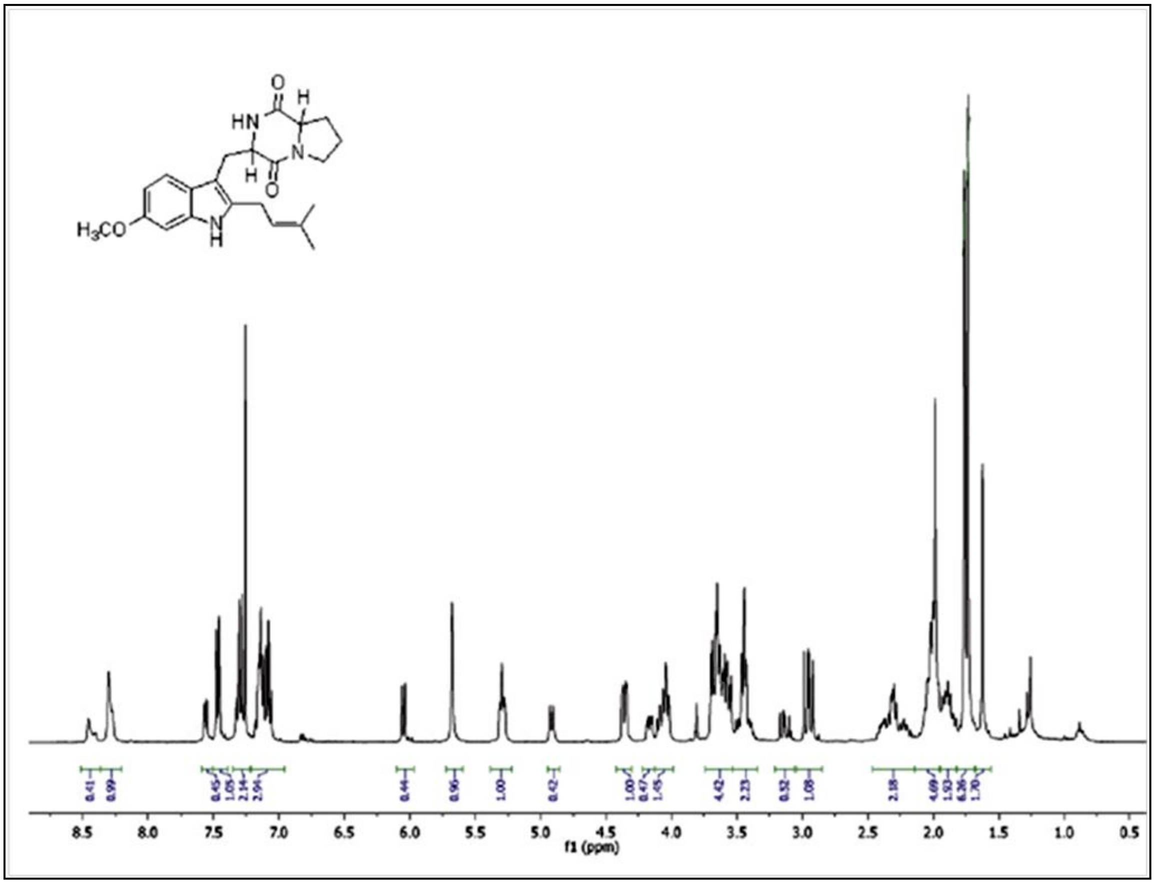

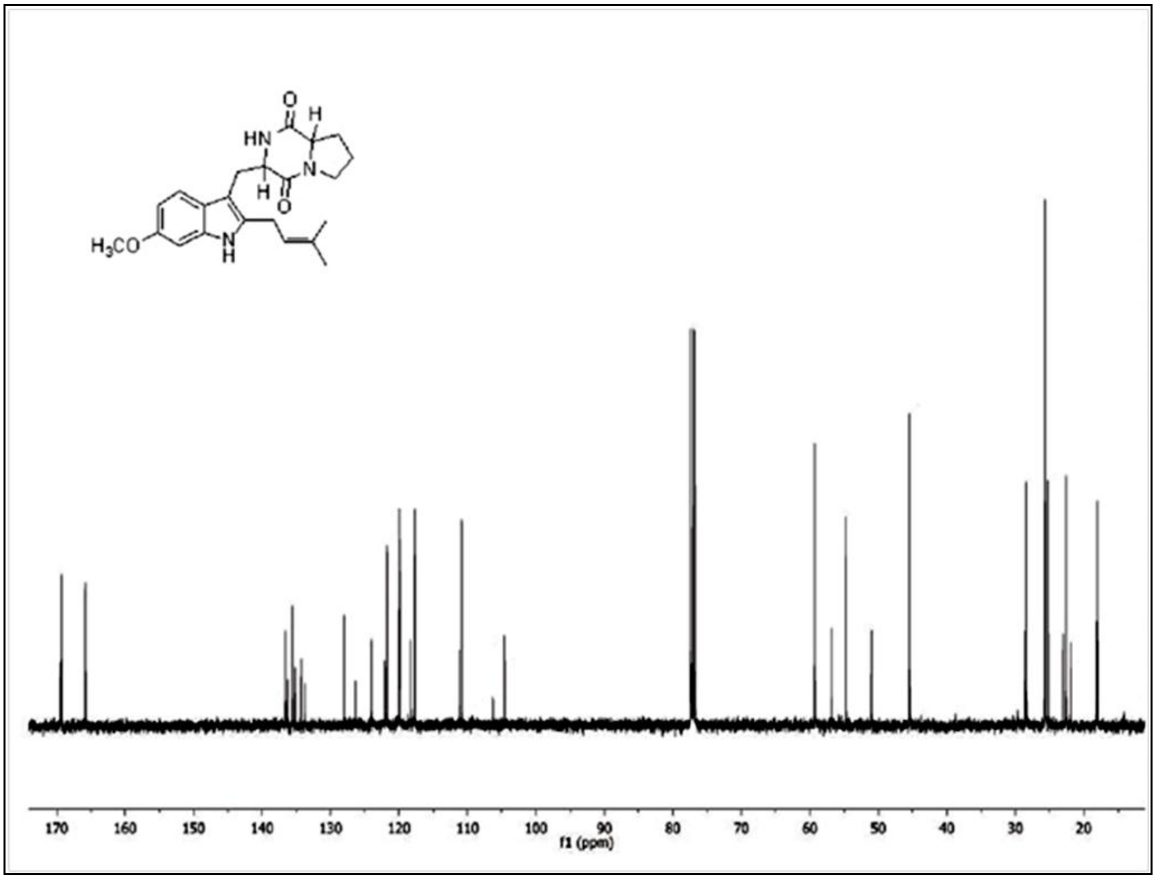

Tryprostatin C: 35% EtOAc:Hexane, 100-200 silica gel for fractionation then prep TLC at 3% MeOH:CH_2Cl_2, Yellow crystalline solid, m.p. 102-104 ^{o}C; $[\alpha]^{25}_{D}$ -48.0 (*c* 0.5, CH_3OH);^{1}H NMR (400 MHz, $CDCl_3$) δ 8.30 (s, 1H, NH), 7.44 (dd, *J* = 17.3, 8.2 Hz, 1H), 7.34 – 7.27 (m, 1H), 7.18 – 7.13 (m, 1H), 7.12 – 7.05 (m, 1H), 5.67 (s, 1H, NH), 5.29 (t, *J* = 7.2 Hz, 1H), 4.36 (dd, *J* = 11.2, 2.6 Hz, 1H), 4.06 (dd, *J* = 15.9, 8.1 Hz, 1H), 3.76 – 3.53 (m, 3H), 3.50 – 3.36 (m, 2H), 2.94 (m, 1H), 2.41 – 2.19 (m, 2H), 2.06 – 1.93 (m, 2H), 1.78 (s, 3H), 1.73 (s, 3H);^{13}C NMR (100 MHz, $CDCl_3$) δ 169.4, 165.8, 136.5, 135.5, 134.2, 128.0, 122.0, 119.9, 118.2, 117.7, 110.8, 104.4, 59.2, 54.7, 45.4, 28.5, 25.7, 25.1, 23.0, 22.6, 17.9; HR-ESI-MS (*m*/*z*) 352.2099 $[M+H]^+$ (calcd for $[C_{21}H_{25}N_3O_2+H]^+$ 352.2025).

Bis(dithiomethyl)gliotoxin

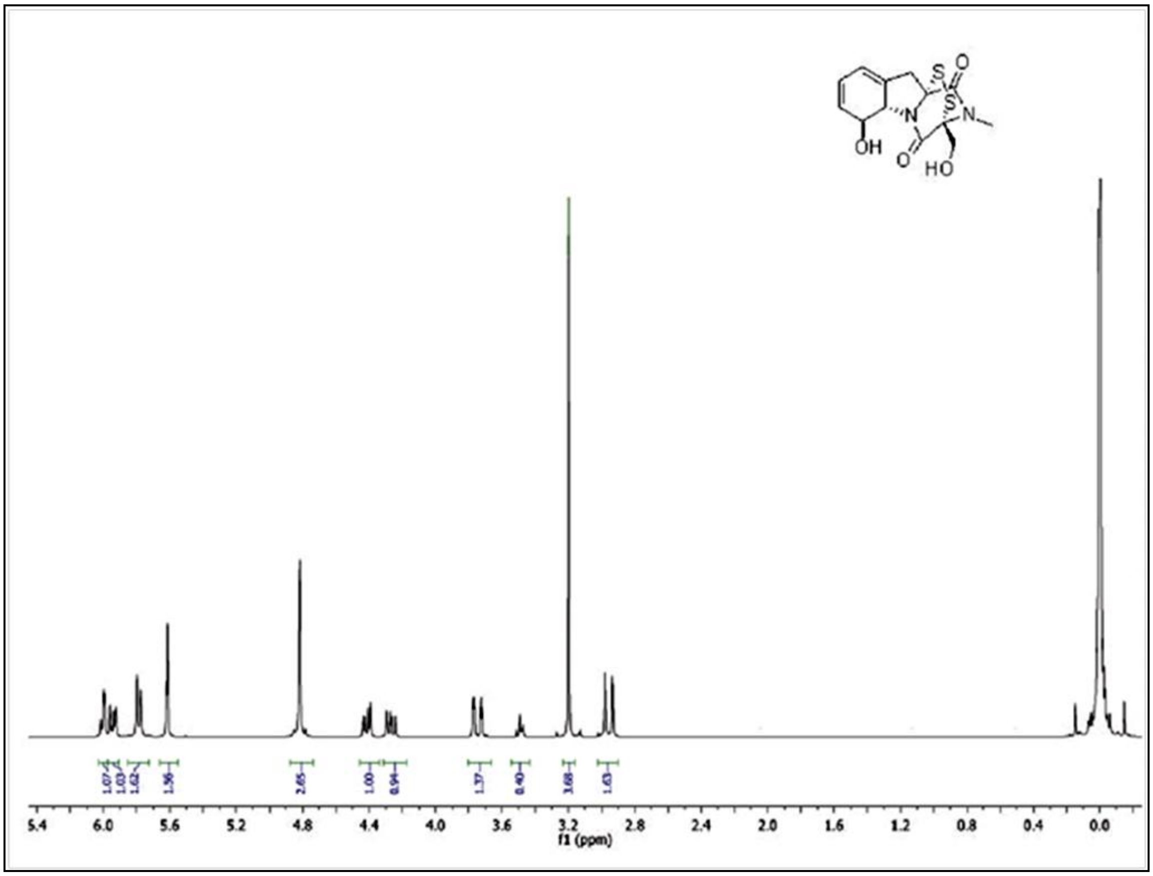

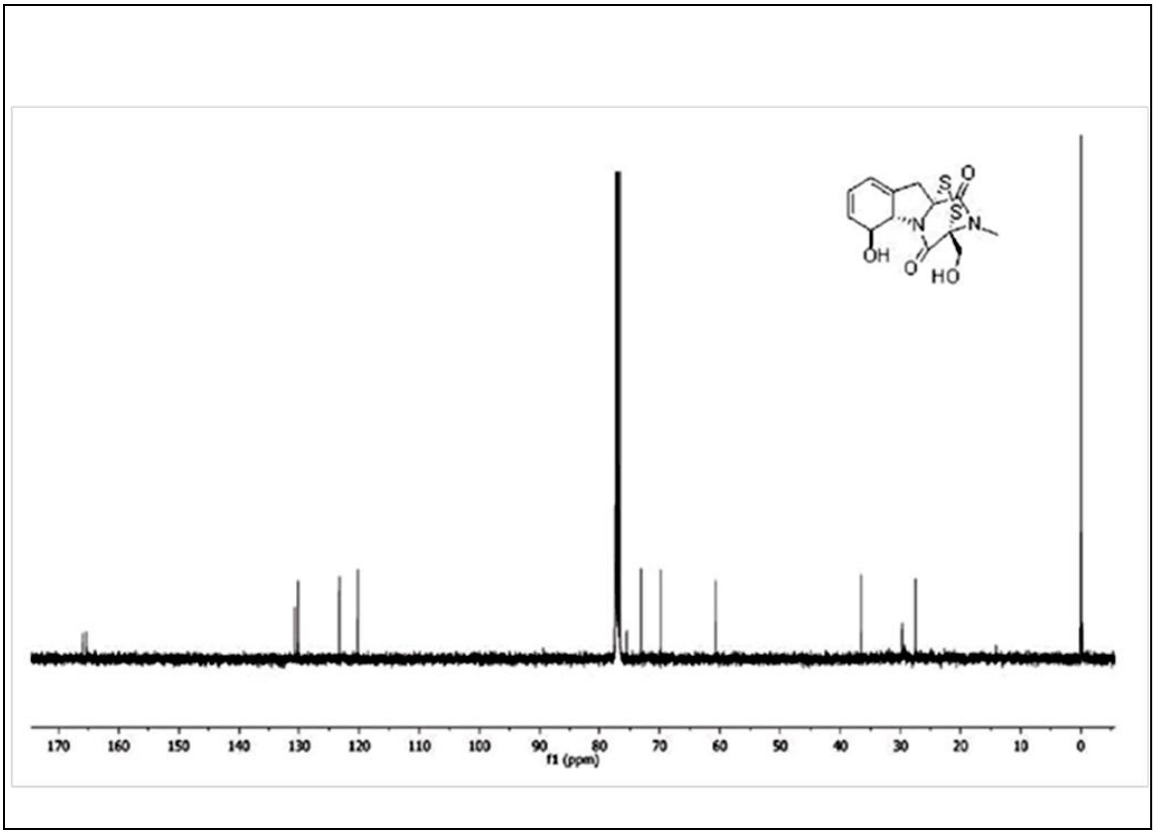

Bis(methylthio)glitoxin: 35% EtOAc:Hexane, 100-200 silica gel,Yellow solid, melting point- 70-71 ^{o}C; $[\alpha]^{25}_{D}$ -23 (*c* 0.5, CH_3OH); ^{1}H NMR (400 MHz, MeOD) δ 5.89 (d, *J* = 2.2 Hz, 1H), 5.87 – 5.79 (m, 1H), 5.58 (d, *J* = 9.7 Hz, 1H), 4.79 (d, *J* = 13.6 Hz, 1H), 4.65 (d, *J* = 13.6 Hz, 1H), 4.15 (d, *J* = 11.5 Hz, 1H), 3.77 (d, *J* = 11.5 Hz, 1H), 3.07 (s, 2H), 3.01 (s, 3H), 2.16 (s, 6H); ^{13}C NMR (100 MHz, MeOD) δ 168.4, 167.8, 134.0, 130.7, 124.8, 120.8, 75.7, 74.3, 73.1, 70.5, 64.6, 39.7, 29.1, 15.2, 13.5; HR-ESI-MS (*m*/*z*) 358.0023 $[M+H]^+$ (calculated for $[C_{15}H_{20}N_2O_4S_2+H]^+$ 357.0943).

Gliotoxin

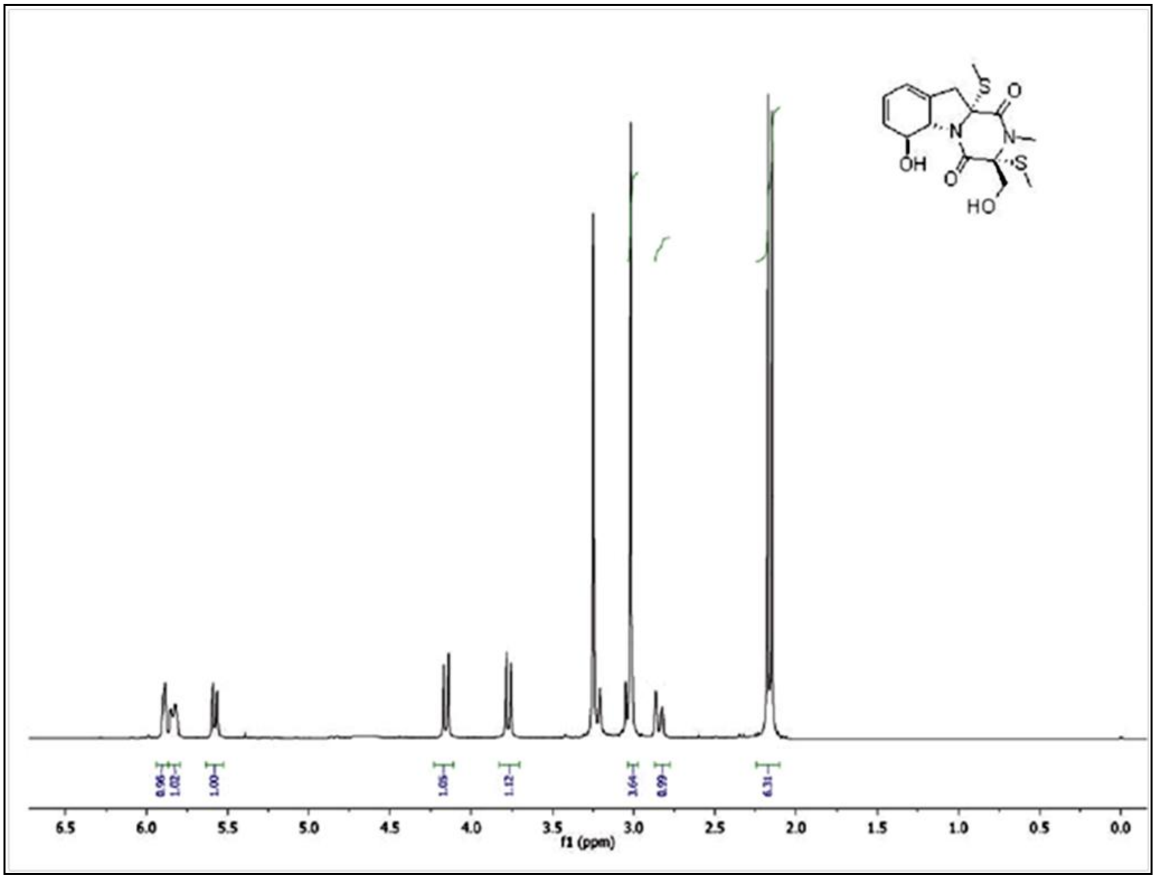

6.5
6.0
5.5
5.0
4.5
4.0
3.5
3.0
2.5
2.0
1.5
1.0
0.5
0.0
f1 (ppm)
0.96
1.02
1.00
1.05
1.12
3.64
0.99
6.31
OH
N
O
S
HO

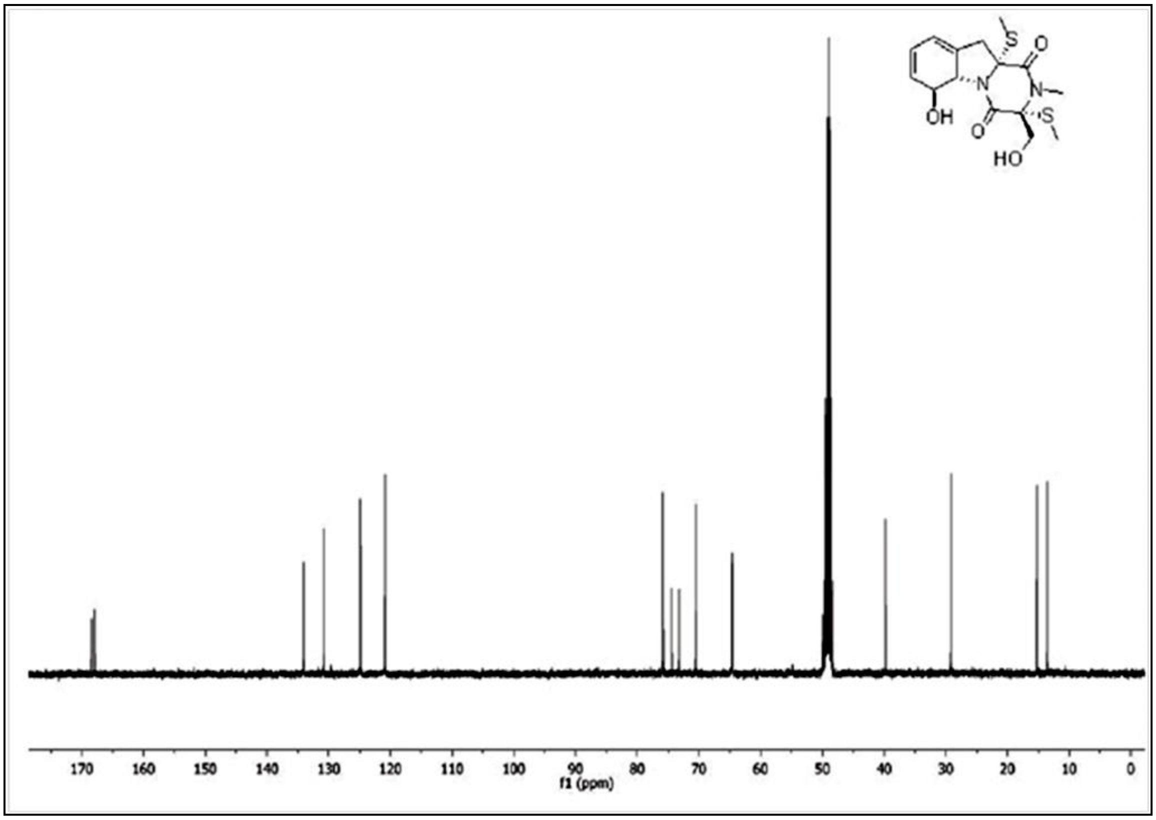

Gliotoxin: 40% EtOAc:Hexane, 100-200 silica gel), Colourless crystalline solid, melting point- 220-222 °C; $[\alpha]^{25}_{D}$ -345 (*c* 0.1, CH_3OH; ^1H NMR (400 MHz, $CDCl_3$) δ 6.00 (d, *J* = 4.8 Hz, 1H), 5.97 – 5.90 (m, 1H), 5.85 – 5.72 (m, 1H), 5.62 (s, 1H, -OH), 4.88 – 4.74 (m, 1H), 4.41 (dd, *J* = 12.7, 5.3 Hz, 1H), 4.27 (dd, *J* = 12.7, 9.3 Hz, 1H), 3.81 – 3.66 (m, 1H), 3.49 (dd, *J* = 10.5, 5.6 Hz, 1H), 3.20 (s, 3H), 3.02 – 2.90 (m, 1H); ^{13}C NMR (100 MHz, $CDCl_3$) δ 166.0, 165.3, 130.7, 130.0, 123.3, 120.2, 75.5, 73.1, 69.8, 60.7, 36.5, 27.4; HR-ESI-MS (*m*/*z*) 349.0307 $[M+Na]^+$ (calculated for $[C_{13}H_{14}N_2O_4S_2+Na]^+$ 349.0293).

Fumiquinazoline C

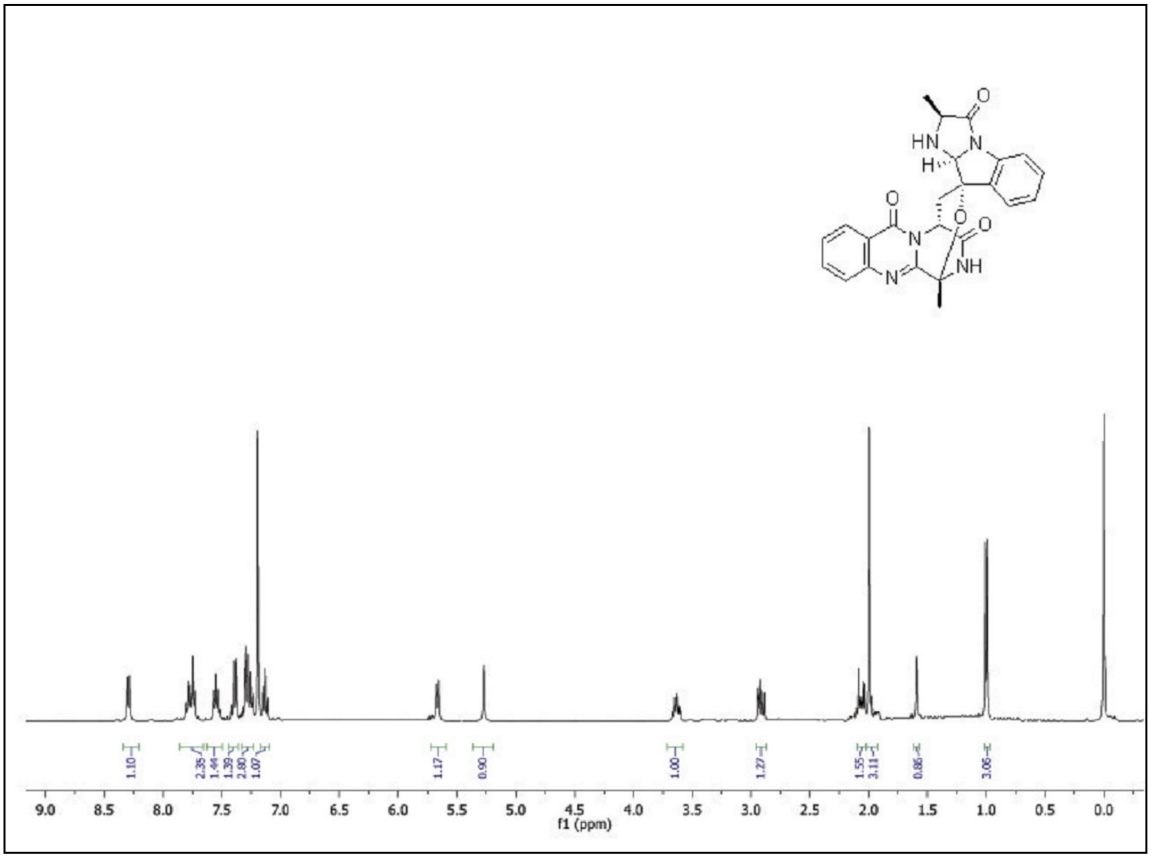

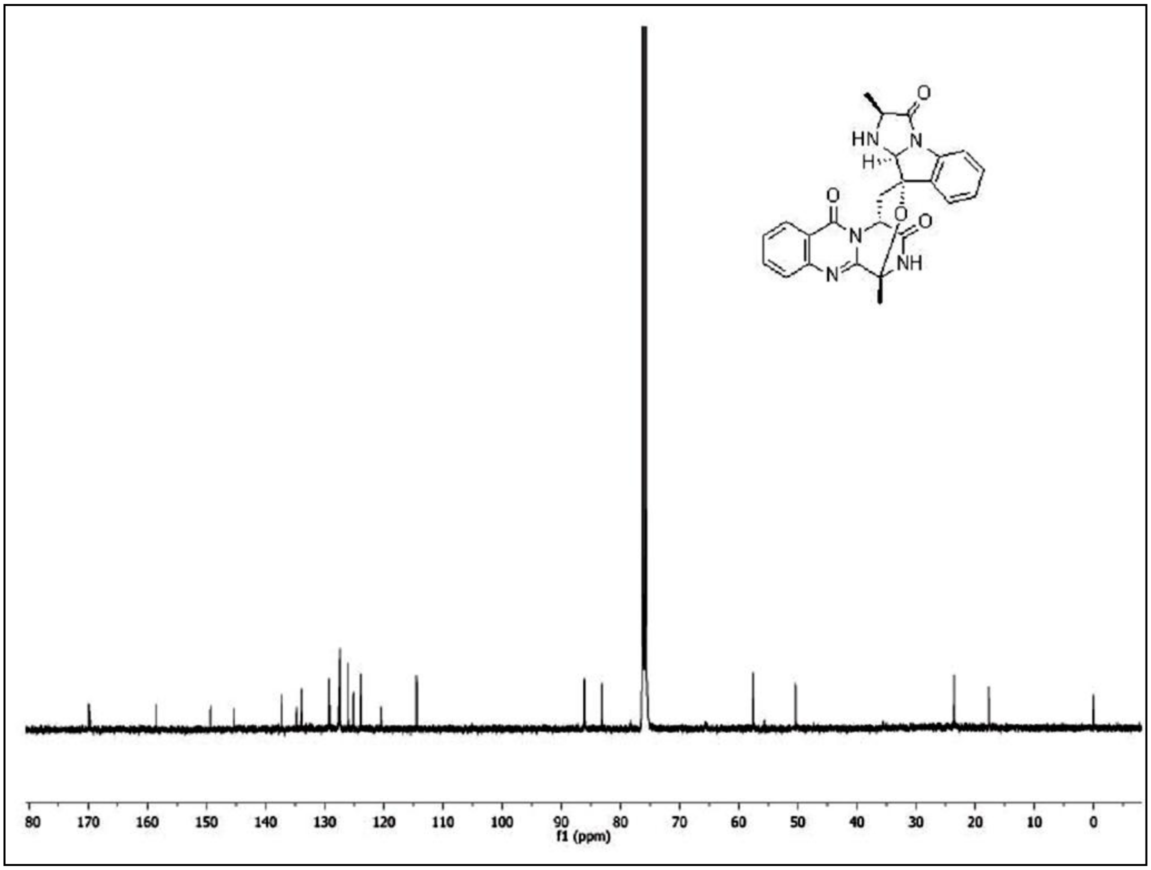

Fumiquinazoline C: 35% EtOAc/Hexane, 100-200 flash silica gel), White solid, melting point- 245-246 ^{o}C; $[\alpha]^{25}_{D}$ -245 (c 0.5, CH_3OH);^{1}H NMR 8.38 (d, J = 7.4, 1H), 7.86(dd, J = 7.4, 6.3, 1H), 7.81 (d, J = 7.4, 1H), 7.62 (dd, J = 7.4, 6.3, 1H), 7.46 (d, J = 7.4, 1H), 7.37 (d, J = 7.9, 1H), 7.32 (dd, J = 7.9, 1.1, 1H), 7.21 (m, 1H), 7.03 (br, NH, 1H), 5.74 (d, J =7.4, 1H), 5.35 (br d, J = 6.7, 1H), 3.71 (dd, J = 6.7, 6.7, 1H), 2.99 (dd, J = 15.3, 7.4, 1H), 2.14 (d, J =15.3, 1H), 2.06 (s, 3H), 1.07 (d, J= 6.7, 3H), 1.03 (s, NH, 1H); ^{13}C NMR (100 MHz, $CDCl_3$) δ 170.8, 170.2, 59.5, 150.3, 146.3, 138.3, 135.7, 134.9, 130.3, 128.6, 128.4,127.0, 126.2, 124.8, 121.4, 115.5, 87.2, 87.0, 84.1, 58.6, 51.4,31.4, 24.6, 18.7; HR-ESI-MS (m/z) 444.1689 $[M+H]^+$ (calculated for $[C_{24}H_{21}N_5O_4+H]^+$ 444.1672).
